# Three recombinantly expressed apple tyrosinases suggest the amino acids responsible for mono- versus diphenolase activity in plant polyphenol oxidases

**DOI:** 10.1038/s41598-017-08097-5

**Published:** 2017-08-18

**Authors:** Ioannis Kampatsikas, Aleksandar Bijelic, Matthias Pretzler, Annette Rompel

**Affiliations:** Universität Wien, Fakultät für Chemie, Institut für Biophysikalische Chemie, Althanstraße 14, 1090 Wien, Austria

## Abstract

Tyrosinases and catechol oxidases belong to the polyphenol oxidase (PPO) enzyme family, which is mainly responsible for the browning of fruits. Three cDNAs encoding PPO pro-enzymes have been cloned from leaves of *Malus domestica* (apple, *Md*PPO). The three pro-enzymes *Md*PPO1-3 were heterologously expressed in *E. coli* yielding substantial amounts of protein and have been characterized with regard to their optimum of activity resulting from SDS, acidic and proteolytic activation. Significant differences were found in the kinetic characterization of *Md*PPO1-3 when applying different mono- and diphenolic substrates. All three enzymes have been classified as tyrosinases, where *Md*PPO1 exhibits the highest activity with tyramine (k_cat_ = 9.5 s^−1^) while *Md*PPO2 and *Md*PPO3 are also clearly active on this monophenolic substrate (k_cat_ = 0.92 s^−1^ and k_cat_ = 1.0 s^−1^, respectively). Based on the activity, sequence data and homology modelling it is proposed that the monophenolase and diphenolase activity of PPOs can be manipulated by the appropriate combination of two amino acids, which are located within the active site cleft and were therefore named “activity controllers”.

## Introduction

Tyrosinases and catechol oxidases are metalloenzymes of the type III copper family and constitute the class of polyphenol oxidases (PPOs)^1, 2^. PPOs contain a di-copper active centre, where each copper ion is coordinated by three conserved histidines^3^. These enzymes are omnipresent among bacteria, fungi^4^, plants^5, 6^ and animals. Some PPOs have been identified as enzymes that participate in anabolic biosynthesis pathways, such as the biosynthesis of the colouring substances betalains and aurones^7–10^, and the biosynthesis of the 8 to 8′ linked lignans in the creosote bush (*Larrea tridentata*)^11^. Catechol oxidases catalyze the two electron oxidation of *ortho*-diphenols to the resultant *o*-quinones coupled to the reduction of molecular oxygen (diphenolase activity, EC 1.10.3.1). Tyrosinases, on the other hand, catalyze the *ortho*-hydroxylation of monophenols to *o*-quinones (monophenolase activity, EC 1.14.18.1)^12, 13^. The products of these reactions are highly reactive and can undergo further non-enzymatic reactions producing complex polymers known as melanins^14, 15^.

In plant cells, PPOs are located in the thylakoid membrane or thylakoid lumen of the chloroplasts with the putative substrates being located in the vacuole^16^. The loss of cell compartmentation, which is accompanied by oxygen penetration due to bruising, wounding or other damage of the plant tissue, could lead to PPO-substrate contact and thus to oxidative reactions^17, 18^. These oxidative browning reactions in plants and the associated formation of melanin after cell wounding are supposed to be a significant defence mechanism^19, 20^. The wound-induced expression of PPOs has been previously described in apples^17^, tomatoes^21^ and potatoes^22^.

*In vivo*, plant PPOs are typically expressed as 54-62 kDa latent pro-enzymes with an N-terminal transit peptide of 8–12 kDa which can be removed proteolytically^6, 23^. The dominant conjecture concerning the enzyme’s activation in plants is a proteolytic processing of the C-terminal domain (15–20 kDa)^20^. However, the latent enzyme can also be activated *in vitro* by proteases (e.g. trypsin, proteinase K)^24, 25^, acidic pH^26^, fatty acids^27^ and detergents (e.g. sodium dodecyl sulfate (SDS))^24, 28^. The use of SDS as an activator is rather unusual for enzymes in general, but PPOs are able to tolerate even very high concentrations of SDS setting them apart from the majority of other enzymes^24, 28, 29^.

Reports regarding the successful heterologous expression of plant PPOs in *E. coli* (or other expression systems) appear inconclusive in the existing literature as there is only a limited number of studies reporting e.g. the expression of insoluble protein fractions^23, 30^, soluble but inactive proteins^31^ or limited amounts of soluble and active recombinant proteins^32–35^. None of these methods has produced high quantities of pure recombinant PPO and therefore are not considered as good protocols for efficient recombinant plant PPO production. PPOs can exist in both the active and inactive (latent) form (with an attached or dissociated C-terminal domain)^24^. However, the mechanism of the *in vivo* activation is so far not understood. In order to prevent folding problems in *E. coli*, the latent form was chosen for heterologous expression in this study. Applying an acidic pH can activate latent PPOs supposedly due to pH-induced conformational changes in the enzyme^26^. In plants, the pH optimum of PPOs varies widely from pH 3.5 to pH 8.0 with apple PPOs exhibiting their optimum value between pH 3.5 and 4.5^19, 36, 37^.

A patent (US20140041079)^38^ has been recently granted for transgenic varieties of non-browning apples (Arctic^®^). The non-browning feature has been introduced by plant genetic engineering using *Agrobacterium tumefaciens* to incorporate a chimeric PPO suppression transgene into the apple genome. The transgene is designed to simultaneously suppress the expression of four members of the apple PPO gene family (Table S1).

In the current research, we have cloned three PPO-encoding genes and were able to produce adequate amounts of pure and active recombinant protein for in-detail biochemical research. The pure *Md*PPO1-3 have been characterized by determining the pH optimum and optimal SDS concentration for activity induction. Moreover, *Md*PPO1-3 were proteolytically activated with three different proteases (trypsin, proteinase K and Nagarse). However, only the reaction of trypsin with *Md*PPO2 resulted in a homogeneous, active protein preparation. Subsequently, we have kinetically characterized latent *Md*PPO1-3 and the active *Md*PPO2 with two monophenolic (tyramine, *L*-tyrosine) and two diphenolic (dopamine, 3,4-Dihydroxy-*L*-phenylalanine (*L*-DOPA)) substrates to provide a basis for the classification of those enzymes as either catechol oxidase or tyrosinase. Several previous studies determined the PPO activity in aqueous solution while some have focused on the detection of PPO-activity based on activity staining with substrates upon SDS-PAGE^39–43^. Here we present data arising from both methods.

The exact mode of action of the PPOs and the associated mechanistic distinction between tyrosinases (mono- and diphenolase activity) and catechol oxidases (only diphenolase activity) is still under debate^44^. There have been some attempts to explain the mono-/diphenolase discrepancy but the high similarity of the active centres of both PPO types does not allow any activity prediction solely based on the PPO’s amino acid sequence. Some amino acids were identified which are putatively crucial for the enzyme’s activity like the “gate keeper”, which is located above the first copper in the active centre (CuA) and in plant PPOs usually present in the form of the sterically demanding amino acid phenylalanine^45^. The gate keeper has recently been described as a stabilizer for the correct substrate orientation through hydrophobic T-shaped π-π interactions between the aromatic rings of the substrate and the gatekeeper phenylalanine^46–49^. Another important residue is the so-called “water keeper”, a conserved glutamic acid, which is located at the active site’s entrance and is assumed to stabilize a conserved water molecule which is responsible for the deprotonation of incoming monophenolic substrates^42, 50^. The combination of this glutamic acid and an asparagine next to the first CuB coordinating histidine (H_B1+1_) has been identified as important for the monophenolase activity of PPOs^51^ and was recently even suggested to distinguish monophenolases from diphenolases^42^. In addition, the amino acid immediately after the second CuB coordinating histidine (H_B2+1_) has previously been shown to influence the ratio of mono- and diphenolase activity in a bacterial tyrosinase^52^. However, the exact role of these residues is not irrefutably established. Based on the results presented herein we propose that the two non-conserved amino acids (the first amino acid after the first CuB coordinating histidine (H_B1+1_) and the amino acid immediately following the second CuB coordinating histidine (H_B﻿2﻿+1_)) which we termed “activity controllers”, together with the gate- and water keeper residues, do strongly influence the activity of PPOs.

## Results and Discussion

### Heterologous expression and protein purification

**Figure 1:**
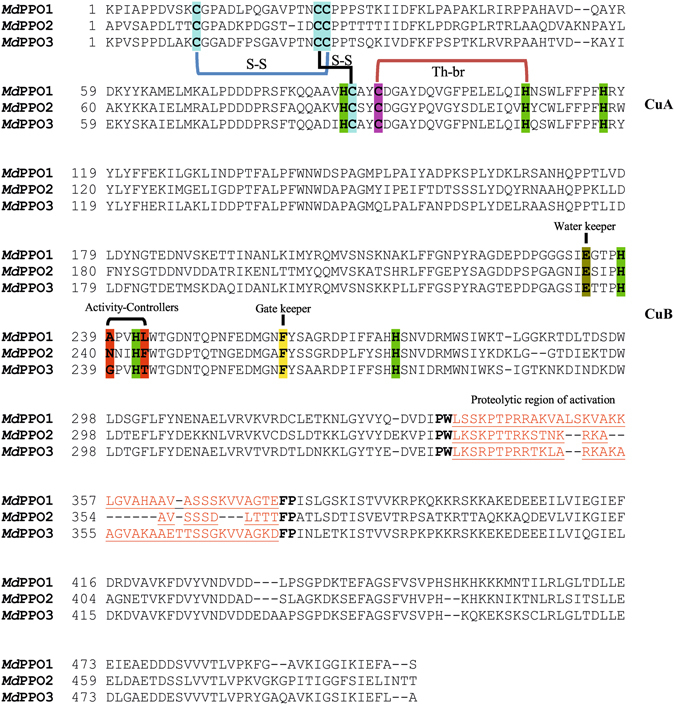
Sequence alignments of the three expressed latent apple tyrosinases (*Md*PPO1-3). Highlighted are the conserved copper-coordinating histidines (green) of the dicopper centre, the conserved cysteines putatively forming disulfide bonds (S-S) (blue), the thioether bridge (Th-br) between a cysteine and the second histidine of CuA (purple-green), the conserved glutamic acid: water keeper (brown), the conserved phenylalanine: gate keeper (yellow), the two amino acids next to the first and the second conserved histidines of CuB: activity controllers (red) and the putative position where the proteolysis activating the latent enzyme takes place (red-underlined).

**Figure 2:**
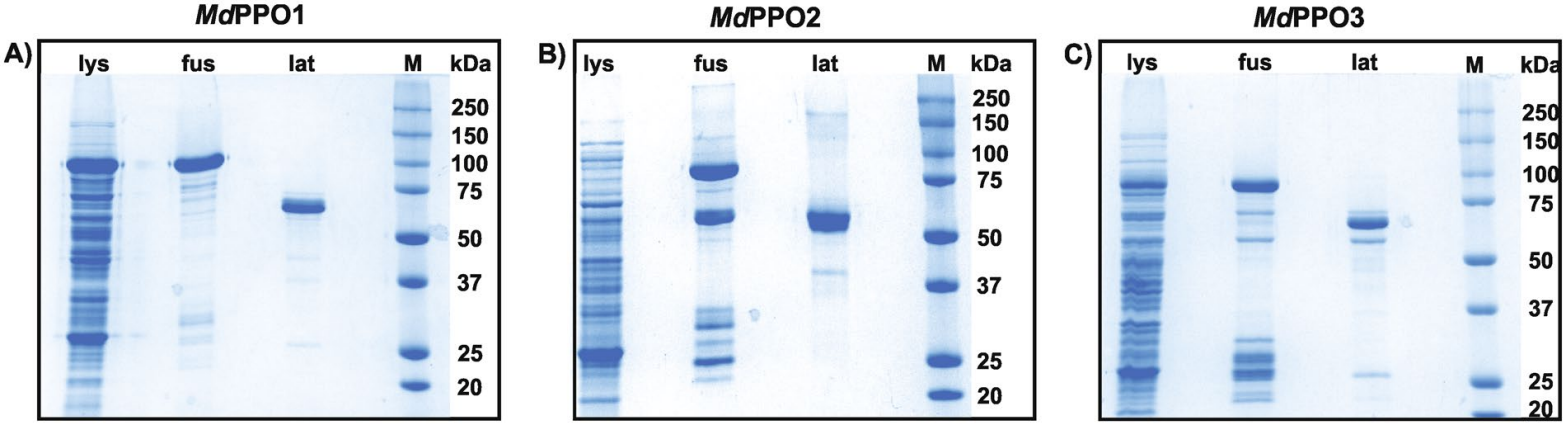
SDS-PAGE gels of *Md*PPO1-3 (**A–C**) at different purification stages. lys): 80 µg of bacterial lysate containing the total soluble protein fraction. fus): 7 µg GST-fusion protein after the first purification step. lat): 7 µg latent PPO after the second purification step. (M): Molecular weight marker. The gels are cropped to the lanes of interest.

Sanger sequencing confirmed the open reading frames in the three *Md*PPO1-3 clones and highlights the conserved motifs as well as similarities between these three enzymes (Fig. 1, Table S5). Sequence verified plasmids were transformed into *E. coli* BL21 for subsequent heterologous expression. The current advanced overexpression method for recombinant plant PPOs in *E. coli* represents a protocol for high-scale production of pure and active PPO. All three *Md*PPO1-3 were expressed soluble in their latent form. The highest yield was obtained for *Md*PPO1 with a final amount of approximately 225 mg per litre of culture. Second in terms of quantity was *Md*PPO2 produced at 60 mg followed by *Md*PPO3 exhibiting the lowest amount with 26.5 mg per litre of culture. All three recombinant *Md*PPO1-3 were produced at yields remarkably higher than any other plant PPOs reported in the literature so far (Table 1). The substantial differences in protein production as compared to previous studies are the use of glutathione-S-transferase (GST) as a fusion partner, the lowered expression temperature of 20 °C and the prolonged expression period of 24–40 hours. The GST tag could act as a chaperone for the folding of the target PPO and allows the fusion protein to be expressed as a soluble protein rather than forming inclusion bodies^53^. The protein expression in *E. coli* growing at 20 °C was chosen in order to improve the solubility, the stability and the correct folding of *Md*PPO1-3. The low temperature could increase the number of chaperones in *E. coli* and reduce the activity of proteases which degrade the overexpressed proteins^54^. The lysis of the bacterial cells seems to be a crucial step as it was noticed that the volume of the lysate should always be determined according to the total bacterial mass in the culture. For the optical densities of the cultures at the time of harvest (7 to 10), a lysate volume of at least 10% of the expression culture’s volume was found to be optimal. Higher concentrations of the lysate could lead to undesirable results like enhanced viscosity and insufficient protein solubility which could reduce the achievable protein yield. The proteins were purified in two steps using a pre-packed 5 ml GSTrap FF column. In the first purification step, the GST-fusion protein was trapped and thereby efficiently captured from the total lysate. During the second purification step, the latent *Md*PPO1-3 was cleaved from the GST-fusion partner and passed through the column as only GST-tagged proteins were trapped. At each purification step (lysate, GST-fusion, latent *Md*PPO) an SDS-PAGE was performed in order to examine the purity and quantity of the proteins (Fig. 2)^55^. Purification tables of the three *Md*PPOs were based on enzymatic activity using the monophenol tyramine as substrate and SDS as activator (Table 2). The purity of the latent *Md*PPO1-3 was at least 95% as indicated by SDS-PAGE. The proteins were stored in 50 mM Tris-HCl pH 7.5 and 200 mM NaCl at 4 °C and were used within 24 h for activity analysis. Overexpression in such high amounts often runs the risk that a major portion of the protein may be present in insoluble fractions, however, investigations of *Md*PPO1-3 expressions showed no significant amount of insoluble fusion protein (Figure S1).

**Table 1:** Heterologous expression yields of plant PPOs per litre of culture.

Plant PPO	yield	reference
PPO (*M. domestica* cv Fuji)	inactive protein	31
PPOs (*Trifolium pratense*)	insoluble protein	30
PPOs (*Camelia sinensis*)	insoluble protein	23
PPO-6 (*Taraxacum officinale*)	0.5–2 mg	32
PPO-2 (*Taraxacum officinale)*	8.7 ± 2.9 mg	33
*cg*AUS1 (*Coreopsis grandiflora*)	5–6 mg	34
*Md*PPO1	225 mg	this work
*Md*PPO2	60.0 mg	this work
*Md*PPO3	26.5 mg	this work

**Table 2:** Purification table for *Md*PPO1-3 from 400 ml of bacterial culture.

Enzyme	Purification step	Protein [mg]	Total activity* [U]	Specific activity* [U mg^−1^]	Yield [%]
*Md*PPO1	Lysate	6400.0	1024.0	0.16	100
	GST-fusion protein	183.6	919.8	5.01	88.1
	Latent PPO	90.0	792.9	8.81	76.0
*Md*PPO2	Lysate	2280.0	118.3	0.05	100
	GST-fusion protein	39.5	24.58	0.62	20.8
	Latent PPO	24.0	16.32	0.68	13.8
*Md*PPO3	Lysate	3032.0	34.00	0.01	100
	GST-fusion protein	26.3	28.67	1.09	84.3
	Latent PPO	10.5	28.14	2.68	82.7

*Determined on 3 mM tyramine with 2 mM SDS.

### Molecular mass determination by ESI-QTOF-MS

Putative masses of the three *Md*PPO1-3 were calculated considering the presence of the two conserved disulfide bonds (−4H) and one thioether bridge (−2H) (Fig. 1). Based on these calculations the three proteins should have the theoretical masses given in Table 3. During the expression process in *E. coli*, it was not clear if the two conserved disulfide bonds and/or the thioether crosslink were successfully formed. The mass spectra of *Md*PPO1-3 (Figure S2) revealed for each PPO a mass that matches with the mass of the protein containing either two disulfide bonds (−4H) and no thioether bridge or only one disulfide bond (−2H) and in addition the thioether bridge (−2H). *Md*PPO1-3 were active and therefore should contain both coppers in their active centres, which are able to autocatalytically form the thioether bridge without special requirements towards the expression host *E. coli* ^56^. Thus, the presence of the thioether bridge is most likely, whereas the presence of the disulfide bond is rather due to the electrospray ionization process as has been reported for free cysteine^57^ than the more unlikely formation of cystine in the *E. coli* cytosol^58^. The same pattern of disulfide bonds and/or the thioether crosslink formation has also been observed for the tyrosinase from the mushroom *Agaricus bisporus* (*ab*PPO4)^59^, walnut tyrosinase (*jr*TYR)^60^ and aurone synthase from *Coreopsis grandiflora* (*cg*AUS1)^10^.

**Table 3:** Masses of *Md*PPO1-3 determined by mass spectrometry.

Enzyme	M (−6H) Da	M (−4H) Da	M (measured) Da	Δ/Da (−4H/measured)
*Md*PPO1	56438.54	56440.56	56440.82	+0.27
*Md*PPO2	57277.68	57279.69	57279.10	−0.60
*Md*PPO3	57504.28	57506.29	57506.72	+0.43

The masses were determined by charge deconvolution of the mass spectra of acidified samples of purified recombinant latent *Md*PPO1-3.

### Characterization of activity induction in latent *Md*PPO1-3

For the three investigated apple *Md*PPO1-3 the optima for acidic activation were determined with the monophenol tyramine as substrate in 50 mM sodium citrate buffer over a pH range from 2.5 to 6 in steps of 0.5 pH units. Figure S3 presents the acidic pH optimum of the three tyrosinases. *Md*PPO1 shows activity between pH 3.5 and 6.0 with the highest activity being exhibited at pH 5.0. *Md*PPO2 has a similar pH optimum at pH 5.0 and the enzyme was active between pH 4.0 and 6.0. *Md*PPO3 seems to be more delicate in this regard since the enzyme’s optimal value was detected at pH 4.0 and it could only be activated between pH 4.0 and 5.0.

The latency of the PPOs is an obstacle during pH optima determination of *Md*PPO1-3. Therefore, we additionally characterized the optimal pH values (as describe above) in the presence of SDS as an external activator and tyramine as substrate. The use of SDS for PPO activation is a widely used and accepted approach. SDS as an activator (in solution^24, 28, 32, 33, 61, 62^, or in SDS PAGE-activity gel reaction^39, 41, 42, 63, 64^) does not change the substrate preference but rather decreases the latency of proenzymes through conformational changes in order to acquire access of the substrate to the catalytic pocket^65, 66^. Figure S4 shows the optimal pH for *Md*PPO1-3 activated by SDS between pH 7.0 and 9.0. In this case, *Md*PPO1 exhibits its optimal activity at pH 7.0, whereas *Md*PPO2 shows higher activity in the range from pH 7.0 to 9.0 with the highest value slightly above pH 7.5, while *Md*PPO3 demonstrates a behaviour similar to that of *Md*PPO1 being most active at pH 7.0. The higher optimal pH values, which were observed during SDS activation, are in accordance with previous results where the behaviour of apple PPO changed with the addition of SDS. In the presence of SDS, the activity of PPOs is inhibited at acidic pH and can only be induced at pH values above 5.0^19^.

The high molarities of SDS, which are necessary to effect PPO activation, make the interactions between the enzyme and SDS particularly interesting to investigate. Thus, Figure S5 shows the optimal SDS concentration for the activation of *Md*PPO1-3 at the enzyme’s respective optimal pH value. *Md*PPO1 was investigated with SDS molarities ranging from 1 to 7 mM in 1 mM steps at pH 7.0 and revealed an optimal SDS concentration of 3 mM. *Md*PPO2 was studied in the same SDS concentration range at pH 7.5 and exhibited optimal activity at 2 mM SDS. The third enzyme *Md*PPO3 appeared to be more tolerant towards higher SDS concentrations as it presented its maximal activity at 4 mM SDS (at pH 7.0) with no significant differences in the concentration range from 2 to 8 mM SDS. To encompass a potential interdependence of the optima in pH value and SDS concentration the two one-dimensional experiments for the determination of optimal pH and optimal SDS concentration were iterated until no significant difference between two consecutive rounds remained.

The latent apple *Md*PPO1-3 have been activated *in vitro* by both exposure to an acidic environment and to high SDS concentrations. It is assumed that both activation methods include conformational changes within the protein leading to its activation^26, 46^. The activities of *Md*PPO1-3 upon SDS activation were more than one order of magnitude higher than those observed upon acidic activation. *Md*PPO1 activation by SDS yielded a maximum activity which was 87-times higher than the one exhibited by acidic activation. In the case of *Md*PPO2 SDS activation yielded a 32-fold higher specific activity and for *Md*PPO3 the SDS induced activity was even 340-times higher than that obtained upon acidic activation. It is obvious that the two modes differ vastly in their effectiveness. The acidic pH seems either to only partially activate the latent enzymes or the enzymes do not tolerate the acidic environment. The SDS activation is more efficient for the three enzymes and also not so detrimental for the enzymes’ stability. The enzymes show a great tolerance towards SDS and are still active at up to 7 and 8 mM of SDS. The mechanism of the SDS induced activation of PPOs is not clearly understood but from our results, it is obvious that acidic pH is not the method of choice for a complete activation of latent apple PPOs^67–69^.

### Substrate specificity of *Md*PPO1-3

As all enzymes were clearly active on tyramine (which is a typical substrate for monophenolase activity) during characterization, *Md*PPO1-3 have to be classified as tyrosinases and are consequently described as tyrosinases in the following. Data on substrate specificity for the three apple tyrosinases *Md*PPO1-3 is outlined in Table 4. We kinetically characterized the three tyrosinases on four substrates, two monophenols (tyramine, tyrosine) and two diphenols (dopamine, *L*-DOPA). In addition we also checked the acceptance of further monophenolic and diphenolic substrates (chemical structures are given in Figure S6). Different PPO isozymes in the same organism raise many questions about the specific target of each enzyme. The natural substrates of each plant PPO isozyme are extremely difficult to identify. However, testing of several substrates can give a good indication of the preferences of each isozyme and the mechanisms of activity.

**Table 4:** Kinetic parameters of the latent *Md*PPO1-3 activated with SDS and the proteolytically activated *Md*PPO2 (*Md*PPO2-act). The entries are given as values ± standard deviation.

Enzyme	Substrate	λ_max_^83^/nm	ε_max_^83^/M^−1^ cm^−1^	K_m_/mM	k_cat_/s^−1^
*Md*PPO1	Tyramine	480	3300	0.71 ± 0.036	9.5 ± 0.43
	Dopamine			0.48 ± 0.038	100 ± 4.54
	*L*-Tyrosine	475	3600	*	*
	*L*-Dopa			3.0 ± 0.30	80 ± 5.2
*Md*PPO3	Tyramine	480	3300	2.4 ± 0.47	1.0 ± 0.71
	Dopamine			2.0 ± 0.16	12 ± 0.67
	*L*-Tyrosine	475	3600	9.4 ± 2.1	0.64 ± 0.12
	*L*-Dopa			19 ± 3.5	6.9 ± 1.2
*Md*PPO2	Tyramine	480	3300	5.6 ± 1.5	0.92 ± 0.19
	Dopamine			5.2 ± 0.91	140 ± 17
	*L*-Tyrosine	475	3600	7.8 ± 4.5	0.15 ± 0.079
	*L*-Dopa			39 ± 12	122 ± 33
*Md*PPO2-act	Tyramine	480	3300	3.5 ± 0.39	0.62 ± 0.051
	Dopamine			3.0 ± 0.43	140 ± 8.5
	*L*-Tyrosine	475	3600	4.9 ± 2.0	0.013 ± 0.0046
	*L*-Dopa			67 ± 23	240 ± 77

*The substrate solubility was too low for a determination of the parameters of the Michaelis-Menten model. Linearization of this model (substrate concentration ≪ K_m_) yielded k_cat_/K_m_ = (0.223 ± 0.0078) mM^−1^ s^−1^.

In the case of monophenolase activity (Table 4), *Md*PPO1-3 show higher specificity for the monophenolic substrate tyramine over *L*-tyrosine. *L*-tyrosine seems to be activated more difficultly as it is converted at the slowest rate by the monophenolase activity of all three apple tyrosinases. On tyramine, *Md*PPO1 has the highest reaction rate (k_cat_ = 9.5 s^−1^) and substrate specificity (K_m_ = 0.71 mM) followed by *Md*PPO3 (k_cat_ = 1.0 s^−1^, K_m_ = 2.44 mM) and *Md*PPO2 (k_cat_ = 0.9 s^−1^, K_m_ = 5.64 mM). *Md*PPO1-3 have also been tested with other monophenolic substrates, specifically (±)-octopamine, tyrosol, phenol, *D*-tyrosine, *L*-tyrosine methylester and *D*-tyrosine methylester. All three enzymes were active on these monophenolic substrates.

Concerning diphenolic substrates (Table 4), *Md*PPO2 shows the highest reaction rate on the substrates dopamine and *L*-3,4-dihydroxyphenylalanine (*L*-DOPA) but also the lowest specificity for those substrates among the three *Md*PPO1-3. *Md*PPO1 shows a very high specificity for the tested diphenols, especially for dopamine. *Md*PPO3 shows higher specificities than *Md*PPO2 but markedly lower reaction rates than the other two enzymes. All of the latent enzymes have lower specificity for *L*-DOPA which was also converted slower than dopamine. *Md*PPO1-3 have also been tested with additional diphenolic substrates and all enzymes were able to oxidise 4-methylcatechol, 4-tert-butylcatechol (TBC), catechol, caffeic acid and 3,4-dihydroxyphenylacetic acid (DOPAC).

Proteolytic activation of the *Md*PPO1-3 using three different proteases did only produce a main activation product in the case of *Md*PPO2 with trypsin. All the other combinations resulted in a band pattern indicative of unspecific digestion (Figure S8). However, partial activation was observed and all three enzymes were active on tyramine, without the need for any additional activator like SDS. Trypsin activation of *Md*PPO1-3 was less efficient than addition of SDS, but more effective than acidic activation. On 3 mM tyramine the mixtures obtained after addition of trypsin (1/50 for 5 minutes) exhibited specific activities that were 33, 4.2 and 31 times lower than the corresponding values attained after addition of SDS for *Md*PPO1, *Md*PPO2 and *Md*PPO3, respectively.

Activation of *Md*PPO2 by limited proteolysis with trypsin (yielding *Md*PPO2-act) appears to increase the enzyme’s specificity for tyramine, dopamine and *L*-tyrosine while lowering it in the case of *L*-DOPA. The maximal reaction rates proved to be very similar for tyramine and dopamine but are slightly different for *L*-tyrosine and *L*-DOPA (Table 4). The order of substrate specificity for *Md*PPO2 is the same for both tested ways of activation revealing that both methods yielded the same pattern of substrate preference.

*Md*PPO1-3 were less specific on tyrosine than on tyramine. This may be due to interactions of the substrates’ carboxylic group with regions of the enzymes’ catalytic pocket. A similar observation was also noted for the PPOs from *Lycopus europaeus* ^70^ and walnut^60^ for the diphenolic substrates containing a carboxylic group such as caffeic acid^71^, DOPAC and *L*-DOPA. *Md*PPO1-3 reveal clear monophenolase activity with greater preference for tyramine instead of tyrosine and diphenolase activity with higher specificity for dopamine than for *L*-DOPA. These results indicate that different isozymes prefer different substrates and thus, *in vivo* each PPO may be responsible for a different set of substrates in order to cover a large spectrum of substrates for various physiological reasons, e.g. pigments formation or defence against herbivores^72, 73^. Thus, the big number of PPO isozymes ceases to be an astounding peculiarity of plants as it may be understood as an effective evolutionary strategy to deal with a varied set of requirements in an effective and yet flexible way.

### The activities of PPOs in aqueous solution and activity gels reveal different perspectives

**Figure 3:**
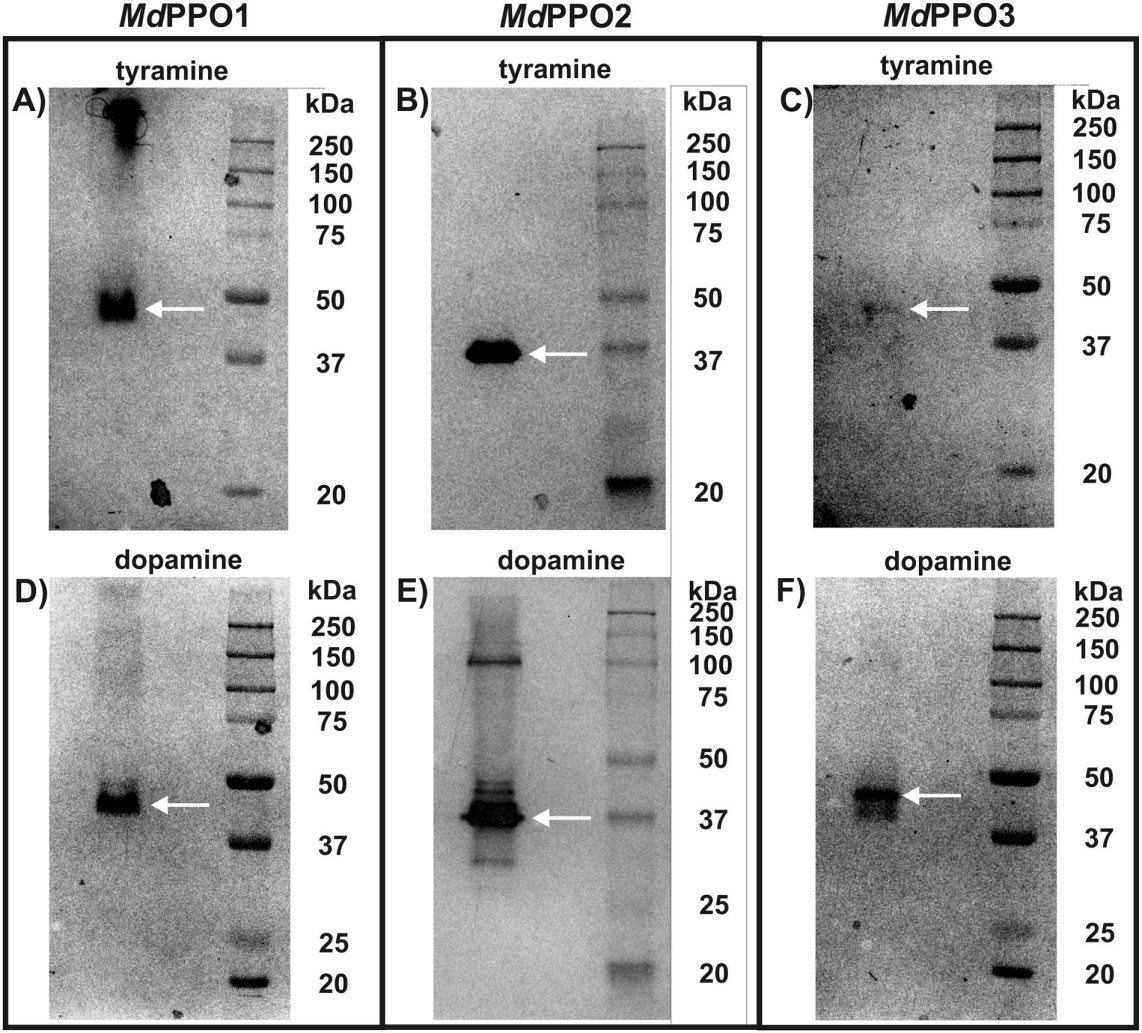
Activity gels of each *Md*PPO1-3 stained with tyramine and dopamine. (**A**) *Md*PPO1 stained with 20 mM tyramine for 30 minutes, (**B**) *Md*PPO2 stained with 20 mM tyramine for 1 minute, (**C**) *Md*PPO3 stained with 20 mM tyramine for 180 minutes, (**D**) *Md*PPO1 stained with 20 mM dopamine for 5 minutes, (**E**) *Md*PPO2 stained with 20 mM dopamine for 15 seconds, (**F**) *Md*PPO3 stained with 20 mM dopamine for 6 minutes. The gels are cropped to the lanes of interest.

Activity staining upon partially denaturing SDS-PAGE was also used to detect the monophenolase activity with tyramine and the diphenolase activity with dopamine of *Md*PPO1-3. This is possible as the enzymes can withstand high concentrations of SDS while still being active on a SDS-gel. Therefore, soaking of the SDS-gels of *Md*PPO1-3 with substrates leads to their conversion under the production of polymerization products which are visible on the gel as dark bands. All three enzymes demonstrated both activities during activity staining after SDS-PAGE (Fig. 3), however, bands appeared after significantly different soaking times depending on the tested substrates. The active bands appeared at positions between 37 and 45 kDa and not at the expected position of the latent forms around 55 to 60 kDa. This is a characteristic phenomenon of latent PPOs in activity gels which still resists explanation^39, 41, 63^.

For *Md*PPO1 the assay with dopamine (diphenolase activity) displayed a band at 45 kDa within 5 minutes. The same enzyme did also react with tyramine (monophenolase activity) and a band was visible after 30 minutes at the same position as for dopamine. *Md*PPO2 was the most robust enzyme in the activity gels as the protein reacted with dopamine yielding a strong band at 37 kDa within 15 seconds and one single band also at 37 kDa with tyramine after one minute. *Md*PPO3 could be characterized as the slowest and consequently the least active in the activity gel among the three enzymes. The enzyme reacted with dopamine in 6 minutes and one band was stained at approximately 45 kDa. The reaction with tyramine was not as vivid and a very faint band appeared around 45 kDa after 180 minutes. Following these results the three *Md*PPO1-3 could be sorted based on their activities according to the time after which a stained band became visible; 1) monophenolase activity based on the substrate tyramine: *Md*PPO2 > *Md*PPO1 > *Md*PPO3 and 2) diphenolase activity based on the substrate dopamine: *Md*PPO2 > *Md*PPO1 > *Md*PPO3. For both mono- and diphenolase activity the sequence of the activity is the same for the three enzymes. In sharp contrast to these results are the conclusions drawn from the reaction rates in aqueous solution with tyramine: *Md*PPO1 > *Md*PPO3 > *Md*PPO2 and dopamine: *Md*PPO2 > *Md*PPO1 > *Md*PPO3 (Table 4).

So far, however, it has not been clearly elucidated whether the two methods provide consistent results for the enzyme activity in order to define a given PPO as either tyrosinase or catechol oxidase. For the first time, we elucidate that the same enzymes show a very different activity behaviour depending whether they are assayed in aqueous solution or in an activity gel. The activity gels (Fig. 3) soaked with the monophenol tyramine indicate that *Md*PPO2 is a very strong tyrosinase (development of a strong band within one minute). On the other hand, the other two isozymes seem to have less or almost negligible monophenolase activity as *Md*PPO1 needs 30 minutes and *Md*PPO3 three hours for the appearance of a faint band. According to these results, *Md*PPO2 and *Md*PPO1 might be called tyrosinases while *Md*PPO3 should be categorized as a catechol oxidase as is not able to create a strong and clear band in the activity gel test with tyramine. In contrast to the activity gels, the reaction rates in aqueous solutions (Table 4) reveal a completely different picture. All of the three apples *Md*PPO1-3 are able to hydroxylate tyramine in a solution containing buffer, substrate, SDS and the respective enzyme. The results in solution classify all isozymes as tyrosinases. Therefore, only activity measurements in aqueous solutions can provide unambiguous information about mono- and diphenolase activity in order to classify an investigated PPO.

### Tyrosinase and catechol oxidase activity are manipulated by amino acid controllers

**Figure 4:**
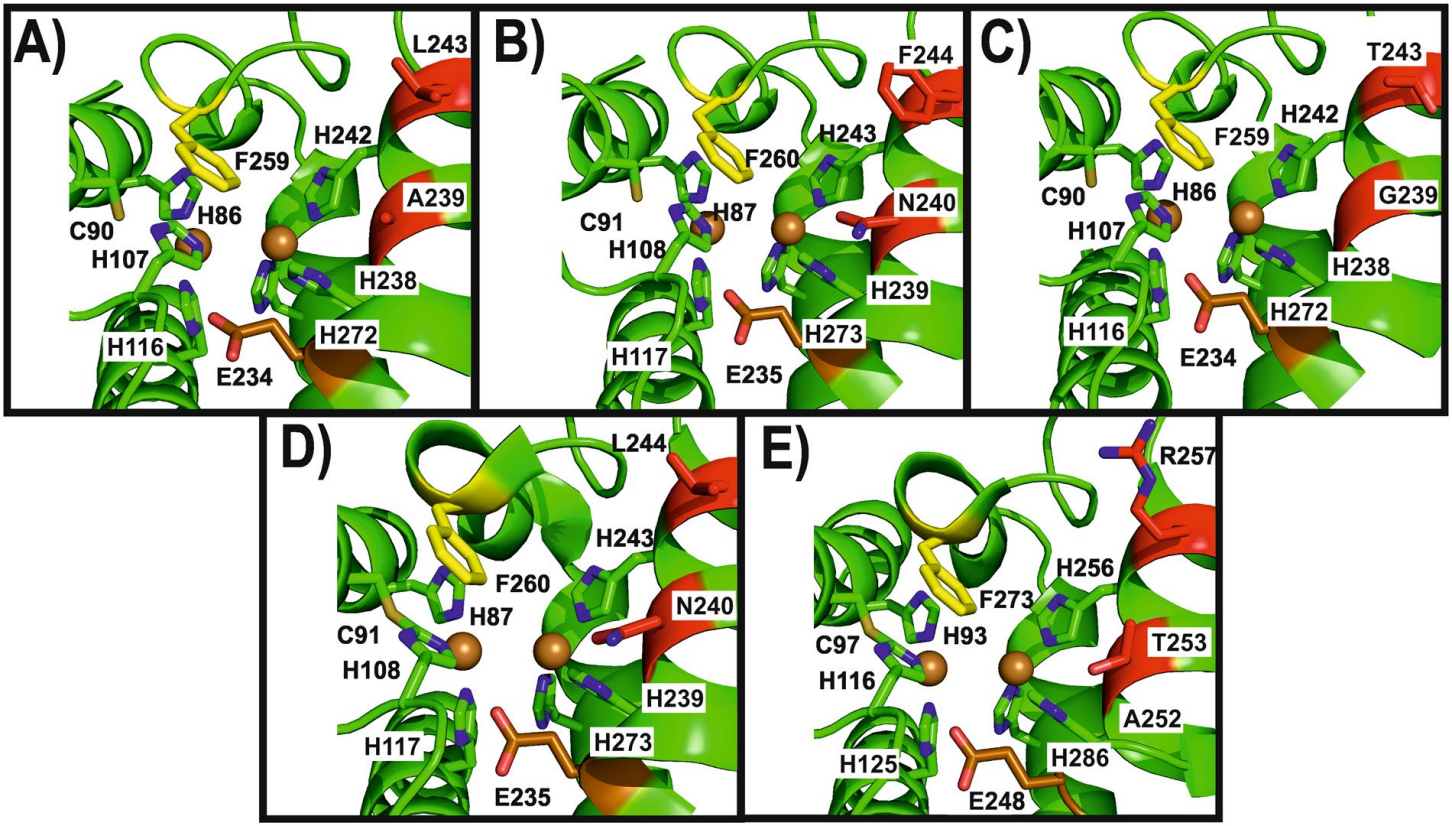
Homology models of the three apple tyrosinases *Md*PPO1-3, *jr*TYR and *cg*AUS1 displaying the respective active site. (**A**) *Md*PPO1, (**B**) *Md*PPO2, (**C**) *Md*PPO3 zoomed into the active centre. (**D**) Active centre of tyrosinase from walnut (*jr*TYR, PDB ID: 5CE9). (**E**) Active centre of the catechol oxidase aurone synthase from *Coreopsis grandiflora* (*cg*AUS1, PDB ID: 4Z11). Highlighted amino acids in all the enzymes: Conserved copper-coordinating histidines (green) coordinated to CuA and CuB (gold spheres), gate keeper (yellow phenylalanine), water keeper (brown glutamic acid) and two activity controllers (red). Nitrogen in the side chains of the highlighted amino acids is displayed in blue.

A diachronic question stagnating for several decades now is why some PPOs have both mono- and diphenolase activity while others are only exhibiting the latter activity. Numerous studies have tried to explain the source of the difference by e.g. crystal structures of different PPOs without definite success as the crystal structures did not reveal any mechanistically significant difference between enzymes commonly classified as either catechol oxidase or tyrosinase^44^. A recent model identified the significance of monophenol deprotonation as a prerequisite for monophenolase activity. More recently, this model was further developed suggesting that one asparagine located next to the conserved first copper-coordinating histidine of CuB could change the activity of a PPO in such a way that it is transformed from a catechol oxidase to a tyrosinase^42, 50^. In order to verify this theory, we compared the active sites of each apple tyrosinase *Md*PPO1-3 generated by homology modelling (Fig. 4A–C). Furthermore, we compared our homology models with the active centres of tyrosinase from *Juglans regia* (*jr*TYR) (Fig. 4D) and catechol oxidase (aurone synthase) from *Coreopsis grandiflora* (*cg*AUS1) (Fig. 4E). The comparison study revealed that the only significant differences between all active sites are the non-conserved amino acids next to the first and second histidine of CuB, whereas the highly conserved gate keeper (phenylalanine above CuA) and the water keeper glutamic acid are located at almost exactly the same position in every structure. The five compared enzymes have different combinations of the above mentioned two non-conserved residues: *Md*PPO1 (Ala239-Leu243), *Md*PPO2 (Asn240-Phe244), *Md*PPO3 (Gly239-Thr243), *jr*TYR (Asn240-Leu244) and *cg*AUS1 (Thr253-Arg257).

**Figure 5:**
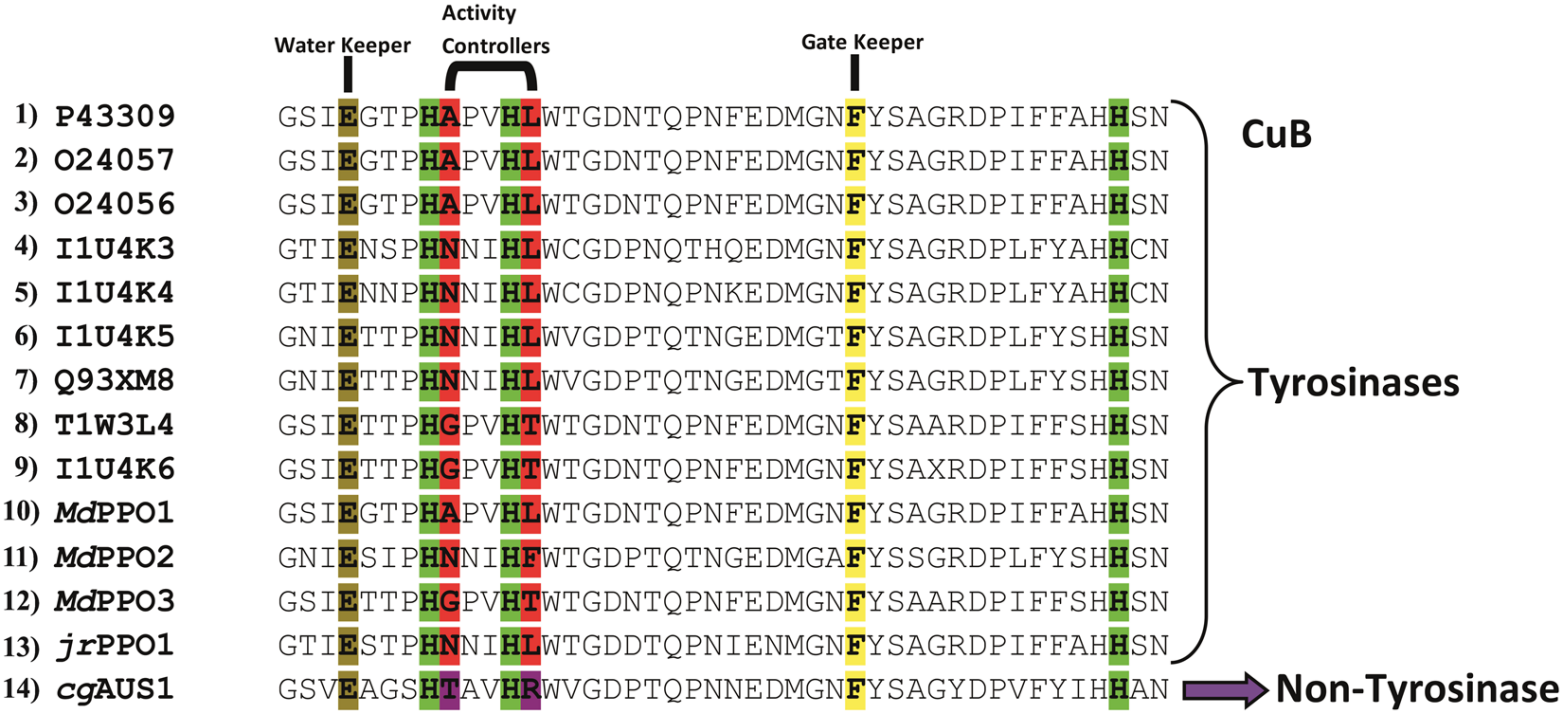
Alignment of PPOs around the CuB region. (1–9) apple PPOs (Uniprot Databank), (10–12) the three recombinant apple tyrosinases *Md*PPO1-3, (13) *jr*TYR and (14) *cg*AUS1. Highlighted are the conserved histidines (green) of CuB, the conserved glutamic acid: water keeper (brown), the conserved phenylalanine: gate keeper (yellow) and the two amino acids next to the first and the second histidine of the CuB which act as activity controllers (red or purple).

Thus according to the asparagine-dependent deprotonation scenario, the only two enzymes with tyrosinase activity should be *Md*PPO2 (Asn240-Phe244) and *jr*TYR (Asn240-Leu244) as only these two enzymes contain the crucial asparagine residue which is putatively necessary for the deprotonation of monophenols. However, we clearly demonstrated that especially *Md*PPO1 but also *Md*PPO3 do clearly possess tyrosinase activity despite lacking the asparagine residue. The only enzyme without tyrosinase activity (based on acceptance of the classical substrates tyrosine and tyramine) in this group is *cg*AUS1 (Thr253-Arg257). Therefore, the “asparagine theory” does not hold for *Md*PPO1 and *Md*PPO3 and we propose that in total four amino acids have the control over tuning the activity of plant PPOs. Namely, the above mentioned highly conserved gate and water keeper and in addition the two non-conserved amino acids, for which we coined the term “activity controllers” based on our results. The gate keeper was shown to act as a stabilizer for the substrate position through hydrophobic T-shaped π-π interactions^46, 48^ and the water keeper seems to stabilize a conserved water molecule which is putatively responsible for the deprotonation of incoming monophenolic substrate^42, 50^. The role of the activity controllers could be the enhancement or attenuation of a monophenolase activity favouring environment depending on the nature and appropriate combination of the activity controllers. The choice and combination of the two amino acids influence the complete environment of the catalytic pocket, hence, the putatively conserved water molecule becomes either basic enough for substrate deprotonation or is not sufficiently activated, which would lead to a lack of monophenolase activity. The presence of negatively or positively charged amino acids in these positions can improve or inhibit the monophenolase activity, respectively. However, also the combination of amino acids with different charges and/or non-charged amino acids could be of great interest in this regard. Figure 5 illustrates the CuB sequence regions of all the putative PPOs from *Malus domestica* that are listed in the Uniprot data bank. According to our theory, the two activity controllers do govern the activity of the PPOs. Applying this model derived from the published substrate preferences of plant PPOs and the *Md*PPO1-3 we assume that all nine (putative) apple PPOs do exhibit tyrosinase activity as the respective activity controllers have the combinations Ala-Leu, Gly-Thr, and Asn-Leu with the first two combinations being present in *Md*PPO1 and *Md*PPO3, respectively, and the third in *jr*TYR. Considering all amino acids for the activity controller positions 400 different possibilities and probably 400 different enzymatic behaviours are possible. Site-direct mutagenesis studies targeted at these two positions might be the stepping stone for the clarification of the actual conditions for tyrosinase activity as well as the source for PPOs engineered to suit specialized applications.

## Methods

Chemicals have been purchased from Sigma-Aldrich (Vienna, Austria) and Carl-Roth (Karlsruhe, Germany) and were at least of analytical grade.

### Plant material, cloning and sequencing of *Md*PPO1-3

Young healthy leaves were obtained from *Malus domestica* cv. Golden delicious apple trees, ground in liquid nitrogen, and the total RNA was isolated by using the RNeasy Plant Mini Kit (Qiagen, Hilden, Germany) according to the manufacturer’s instructions. cDNA was synthesized using the SMARTer^®^ RACE cDNA Amplification Kit (Clontech, Saint-Germain-en-Laye, France). Specific primers (Table S1) for the three apple PPO genes (PPO2, GPO3 and APO5) were designed (omitting the sequence coding for the transit peptide) and used to amplify the three PPO genes from the cDNA template with Q5^®^ High–Fidelity DNA polymerase (NEB, Ipswich, England). PCR products were cloned into the pGEX-6P-1 expression vector (GE Healthcare, Freiburg, Germany) as follows: Once obtained, the amplified PPO genes were phosphorylated with T4 Polynucleotide kinase (NEB) and mixed with the pGEX-6P-1 vector that had been digested with the *Sma*I restriction enzyme (Thermo Fisher scientific, Massachusetts, USA) and dephosphorylated with Calf Intestinal Alkaline Phosphatase (NEB) to prevent religation of the linearized plasmid-DNA. The resulting mixtures were ligated with T4 DNA ligase (NEB) and transformed into chemically component *E. coli* TOP10 cells (Thermo Fisher scientific)^74^. The clones were sequenced externally by Microsynth GmbH (Vienna, Austria).

### Heterologous expression and purification of recombinant PPOs by Fast Protein Liquid Chromatography

*Md*PPO1-3 genes were N-terminally fused with the GST-tag of the pGEX-6P-1 vector. Between the two fusion-partners, the human rhinovirus 3 C protease (HRV3C) recognition sequence (LEVLFQ|GP) is located which allows the controlled dissociation of the two proteins. The three fusion genes (GST-*Md*PPO1-3) were efficiently overexpressed using the synthetic *tac* promoter of the pGEX-6P-1 vector. *E. coli* was grown in a modified 2xYT medium (1.6% tryptone-peptone, 1% yeast extract, 1% NaCl, 0.5% NH_4_Cl, 0.5% glycerol, 2 mM MgCl_2_, 1 mM CaCl_2_ pH 7.5) supplemented with ampicillin (100 µg/ml). The three *Md*PPO1-3 *E. coli* BL21 expression batches were inoculated with saturated overnight cultures and were grown at 37 °C under shaking for 4 hours until the OD_600_ reached a value between 0.6 and 0.8. Afterwards, the temperature was reduced to 20 °C and the cultures were induced with 0.5 mM isopropyl β-D-1-thiogalactopyranoside and 0.5 mM CuSO_4_. The expression cultures remained at 20 °C under shaking for 24 to 40 hours. When the OD_600_ reached a value of 7 to 10 the cultures were collected by centrifugation at 10000 × g for 25 minutes at 4 °C.

Lysis of the cells was performed by the freeze-thaw technique using liquid nitrogen. The pellets were resuspended in a volume of 10% of the original expression culture of lysis buffer (50 mM Tris-HCl pH 7.5, 200 mM NaCl, 1 mM EDTA, 50 mM sucrose). Lysozyme (0.5 g/l) and protease inhibitors (1 mM phenylmethylsulfonyl fluoride and 1 mM benzamidine) were added and the resulting suspensions were incubated for 45 minutes under shaking on ice. Subsequently, the solutions underwent five cycles of freezing in liquid nitrogen and thawing in a water bath at 20 °C. Eventually, 2 mM MgCl_2_ and 0.02 g/l DNaseI were added to the lysates and incubated for 15 minutes at 100 rpm and 25 °C. The lysates were centrifuged at 10000 × g for 1 hour at 4 °C.

The chromatographic purifications were carried out using an Äkta Purifier (GE Healthcare) placed in a refrigerator at 4 °C. The filtrated lysates were placed in a 50 ml injection loop and applied to a prepacked 5 ml GSTrap FF column using 50 mM Tris-HCl pH 7.5, 200 mM NaCl as the binding buffer. Following the trapping and flushing out of unbound proteins, the target proteins were eluted with 50 mM Tris-HCl pH 7.5, 200 mM NaCl and 15 mM reduced glutathione. The GST-fusion protein fraction was concentrated using a Vivaspin ultrafiltration device with a 30 kDa molecular weight cut-off (VWR). The buffer was exchanged to 50 mM Tris-HCl pH 7.0, 150 mM NaCl, 1 mM EDTA and the samples were mixed with GST-HRV3C produced in-house according to^25^ at a mass ratio of 1 to 50 (fusion protein to protease). The proteolysis was carried out over 48 hours at 4 °C. The cleaved protein was then again applied to a 5 ml GSTrap FF column, whereby the GST protein and the GST-tagged protease were still trapped by the column while the latent PPOs passed through the column and eluted immediately in the flowthrough (Figure S7). The protein concentrations were determined according to the Lambert-Beer law and their absorption at 280 nm using the extinction coefficient provided by ExPASy ProtParam^75, 76^.

### Homology modelling of *Md*PPO1-3

The amino acid sequences of the latent forms of *Md*PPO1-3 were submitted to the SWISS-MODEL Workspace^77, 78^, a fully automated protein structure-homology modelling server. As a first step, the pipeline searched for the appropriate template for each PPO type based on BLAST^79^ (sequence alignment algorithm) and HHblits^80^ (sequence-based protein function and structure prediction). The template search resulted in the same best template for all *Md*PPO1-3, namely aurone synthase from *Coreopsis grandiflora* (PDB entry: 4Z11) which exhibited the highest coverage value (0.97 for *Md*PPO1 with 47.5% sequence identity, 0.99 for *Md*PPO2 with 45.2% sequence identity and 0.97 for *Md*PPO3 with 47.4% sequence identity). The final homology models of each *Md*PPO1-3 type were then created using the aurone synthase structure as the template applying the modelling engine PROMOD3^81^. All visualizations were created using the PyMol Molecular graphic system (Schrödinger, LLC) version 1.3^82^.

### Molecular mass determination by ESI-QTOF-MS

Electrospray Ionization Mass Spectrometry (ESI-MS) was performed on a nano electrospray ionisation – quadrupol and time-of-flight mass spectrometer (ESI-QTOF-MS, MaXis 4 G UHR-TOF, Bruker) with a mass range of 50–20000 m/z. Pure latent enzyme at a concentration of 10 g/l was used. The buffer was exchanged to 5 mM ammonium acetate pH 7.0 and the enzyme solution was diluted to 1% (v/v) in 2% acetonitrile and 1‰ formic acid immediately before being applied to the mass spectrometer.

### Characterization of *Md*PPO1-3

The three latent isozymes, *Md*PPO1-3, were characterized by their pH dependence as well as their optimum for activation by SDS or by acidic pH. Monophenolase specific activities with 3 mM tyramine as the substrate were determined spectrophotometrically at 25 °C by measuring the appearance of coloured products at 480 nm (ε_480_ = 3300 M^−1^ cm^−1^)^83^. The optimum for activation induced by acidic pH was determined in 30 mM sodium citrate buffer between pH 2.5 and 6.0 in steps of 0.5 pH units. The pH optimum based on enzymatic activity was analyzed in a range between pH 3.0 and 10.0 in steps of 1.0 pH unit with 2 mM SDS as activator and was repeated at the optimal SDS concentration once this value was established. The measurements were performed in 50 mM sodium citrate buffer for the pH range of 2.0–7.0, in 50 mM Tris-HCl buffer for the pH range of 7.0–9.0 and in 50 mM glycine buffer for the pH range of 9.0–10.0. The optimal SDS concentration for activity induction was analyzed in a range from 1 to 8 mM SDS in 1 mM steps at the priorly determined optimal pH for every *Md*PPO1-3. Experiments were performed in triplicate in a 96 well microplate. Absorption-time curves were recorded on a TECAN infinite M200 (Tecan, Salzburg, Austria).

### Proteolytic activation of *Md*PPO1-3

Conversion of latent *Md*PPO1-3 into active PPOs have been investigated by treatment with three different proteases (trypsin, proteinase K and Nagarse). Proteolytic digestions of *Md*PPO1-3 were investigated applying different ratios of protease and latent PPO as well as different incubation times but homogeneity of the active enzyme was only attained for *Md*PPO2 with trypsin (Figure S8). Hence *Md*PPO2 was proteolytically activated by using trypsin at a ratio of 1:40 (10 µg of trypsin per 1 mg of *Md*PPO2) in 300 mM Tris-HCl pH 8.4 and 100 mM sodium ascorbate at 25 °C for 10 minutes in a total volume of 30 ml containing 12 mg of latent *Md*PPO2. The reaction solution was concentrated to a volume of 200 µl by ultrafiltration (Vivaspin^®^ 20 30 kDa molecular weight cut off). The “digested” solution was applied on a size exclusion column (Superdex 200 Increase from GE Healthcare). Eluted fractions possessing diphenolase activity with 1 mM dopamine (Figure S9) were pooled and concentrated by ultrafiltration (Vivaspin^®^ 500 30 kDa molecular weight cut off). Purity of the activated *Md*PPO2 was checked by SDS-PAGE (Figure S10) indicating a single protein species. Protein concentrations were determined according to the Lambert-Beer law and their absorption at 280 nm using the extinction coefficient provided by ExPASy ProtParam^75, 76^.

### Enzyme kinetics and enzyme activity assays of *Md*PPO1-3

The pH and SDS optimum for each latent *Md*PPO1-3 were applied for the determination of the enzymatic activities. Activity was determined spectrophotometrically by detecting the appearance of the chromophore quinone products from tyrosine, tyramine, dopamine and *L*-DOPA for the determination of the kinetic parameters of the latent *Md*PPO1-3 and the active *Md*PPO2. Absorption curves and spectra were recorded at 25 °C in a 96 well microplate on a TECAN infinite M200 (Tecan). Kinetic measurements were done in a total volume of 200 µl, containing 50 mM Tris-HCl buffer (pH 7.0 for *Md*PPO1 and *Md*PPO3 or pH 7.5 for *Md*PPO2), different molarities of substrates, different molarities of the enzymes and SDS in the case of the latent *Md*PPO1-3 (2 mM for *Md*PPO2, 3 mM for *Md*PPO1 and 4 mM for *Md*PPO3).

Additionally the acceptance of the monophenolic ((±)-octopamine, tyrosol, phenol, *D*-tyrosine, *L*-tyrosine methyl ester and *D*-tyrosine methyl ester) and diphenolic substrates (catechol, 4-methylcatechol, 4-tert-butylcatechol (TBC), caffeic acid and 3,4-dihydroxyphenylacetic acid (DOPAC)) were determined for the three apple tyrosinases. The molar absorption coefficients (ε_λmax_) of the formed chromophores have already been reported in the literature for a number of phenolic substrates (Table S2)^83^. The coefficient values for the remaining diphenols were determined in 50 mM Tris-HCl buffer at pH 7.0 with NaIO_4_ as the chemical oxidant as described by Muñoz *et al.* ^83^. Absorption coefficients for the monophenols were determined in a similar way but the oxidation was carried out enzymatically with tyrosinase. Various amounts of substrate (in the µM range) were oxidised chemically with an excess of NaIO_4_ or enzymatically in the case of monophenols and the formation of quinones was monitored spectrophotometrically. The molar extinction coefficient was then determined by linear regression at the appropriate wavelength (λ_max_) (Figure S11). Spectra were taken routinely for each substrate on a Shimadzu UV-1800 spectrophotometer (Shimadzu Deutschland, Duisburg, Germany) in 1 ml of solution at 25 °C.

### Activity staining after SDS-PAGE and SDS tolerance of *Md*PPO1-3 in the activity gel

All three latent *Md*PPO1-3 have been used for activity detection and comparison on SDS-PAGE activity gels with one monophenolic (tyramine) and one diphenolic (dopamine) substrate. 25 µg of each *Md*PPO1-3 were mixed with loading buffer lacking SDS and β-mercaptoethanol and applied without heating to a partially denaturing 11% SDS-PAGE gel^64^. For the activity-staining, the gels were soaked in 20 mM tyramine or 20 mM dopamine, respectively, at optimum conditions regarding pH and SDS concentrations for each enzyme (which were previously determined in solution). The time until the appearance of dark bands was measured for each enzyme (Fig. 3). Moreover, a similar procedure was performed by mixing 25 µg of each *Md*PPO1-3 with loading buffer in absence of β-mercaptoethanol but with different molarities of SDS (1.5, 4, 7.5, 19, 37, 56, 75, 150 mM). For the staining, gels were soaked in 20 mM dopamine at optimum conditions. The different molarities of SDS in the loading buffer were investigated for an effect on the activity of the latent *Md*PPO1-3 enzymes after SDS-PAGE (Figure S12).

## Electronic supplementary material

Supplementary Information
