# Supplementary material for: Three recombinantly expressed apple tyrosinases suggest the amino acids responsible for mono- versus diphenolase activity in plant polyphenol oxidases
Source: Sci Rep. 2017 Aug 18;7:8860. doi: 10.1038/s41598-017-08097-5 (PMC5562730; doi:10.1038/s41598-017-08097-5)
Supplement: Supplementary file 1 — Supplementary Information [file 41598_2017_8097_MOESM1_ESM.doc]

**Supplementary Information**

**Three recombinantly expressed apple tyrosinases suggest the amino acids responsible for mono- versus diphenolase activity in plant polyphenol oxidases**

Ioannis Kampatsikas^1^, Aleksandar Bijelic^1^, Matthias Pretzler^1^ & Annette Rompel^1*^

^1^ Universität Wien, Fakultät für Chemie, Institut für Biophysikalische Chemie, Althanstraße 14, 1090 Wien, Austria; https://www.bpc.univie.ac.at

*Correspondence to: annette.rompel@univie.ac.at

**Methods**

**Soluble and insoluble fractions of *Md*PPO1-3.** The heterologous overexpression in *E. coli* of *Md*PPO1-3 yielded high amounts of recombinant protein (Table 1). The insoluble fractions were investigated by SDS-PAGE in order to clarify whether a part of the *Md*PPO1-3 would be targeted to inclusion bodies or other insoluble protein fractions. The *Md*PPOs1-3 were expressed soluble and no significant portion of insoluble *Md*PPO1-3 was detected (Figure S1).

**Cloning and sequencing analysis of *Md*PPO1-3.** Cloning attempts with primers (Table S1) designed from the three apple PPO sequences L29450, AF380300 and KF032055 were successful. The primers and the expression constructs were designed and created in order to produce the latent pro-enzyme of each *Md*PPO1-3 as an N-terminal fusion protein with GST. Sequencing of the three cDNA clones *Md*PPO1 (LT718522), *Md*PPO2 (LT718523) and *Md*PPO3 (LT718524) showed minor differences from the prototype sequences used to design the primers APO5 (L29450), PPO2 (AF380300) and GPO3 (KF032055). *Md*PPO1 is almost identical to the APO5 (L29450) template with only one silent mutation in the sequence. *Md*PPO2 and *Md*PPO3 have specific mutations and could be denominated as isozymes of PPO2 (AF380300) and GPO3 (KF032055), respectively (Table S3). The three encoded proteins *Md*PPO1-3 did present a high level of differences in their amino acid sequences with *Md*PPO1 being closer to *Md*PPO3 and *Md*PPO2 being most different from the other two enzymes (Table S4). The cloned *Md*PPO1-3 genes were predicted to encode 3 PPO-pro-enzymes. The latent enzymes contain 505, 495 and 507 amino acids for *Md*PPO1, *Md*PPO2 and *Md*PPO3, respectively (Table S5).

**Calculation of absorption coefficients.** The molar absorptions of the chromophores produced from the diphenol 3,4-dihydroxyphenylacetic acid (DOPAC) and the monophenols (±)-octopamine, tyrosol, L-tyrosine methyl ester and D-tyrosine methyl ester were calculated by generating the quinone products through oxidation with an excess of periodate (NaIO_4_) for the diphenol (DOPAC) or oxidation with tyrosinase for the monophenols. The appearance of corresponding quinones was monitored at specific wavelengths for every substrate. The quinones formation was monitored spectrophotometrically at several time points. The extinction coefficients were determined by linear regression of the substrate concentration versus the measured absorption at the specific wavelength (Figure S11). ^1^

**Activity stained SDS-PAGE with different molarities of SDS.** *Md*PPOs1-3 were studied by partially denaturing SDS-PAGE activity gels with 20 mM dopamine and different molarities of SDS (Figure S12). The loading buffer contained different molarities of SDS ranging from 1.5 mM to 150 mM and were incubated with the samples for 5 minutes at room temperature before gel loading. *Md*PPO1 was active up to an SDS concentration of 100 mM but starting from 56 mM SDS the bands were notably fainter. Two bands were visible on the gel, one at approximately 45 kDa and one around 70 kDa. *Md*PPO2 showed great tolerance towards SDS and showed one band on the gel at 37 kDa. *Md*PPO3 was similarly active after incubation with all the tested molarities of SDS in the loading buffer and two close bands appeared between 40 and 45 kDa. As in the activity gels lacking SDS in the loading buffer the activity band did always appear in a position different from that of the latent protein (55-60 kDa). Moreover, even higher concentrations of SDS did not inhibit the formation of a stained band but did only modify the result very slightly from the picture seen without SDS in the loading buffer (Figure 3).

# Tables

**Table S1: Data base sequences and primers used to amplify the expressed genes.**

| **PPOs** | **Primers** | **Accession number** | **Size [bp]** | **ORF [AA]*** | **MW [kDa]** |
| --- | --- | --- | --- | --- | --- |
| APO5 | fw 5΄AGCCTATAGCCCCACCAGACG 3΄  rev 5΄CTAAGAAGCAAATTCAATCTTGATACCACCAA 3΄ | L29450 | 1515 | 505 | 56.2 |
| PPO2 | fw 5΄AGCACCGGTATCCGCC 3΄  rev 5΄TTAGGTAGTATTAATGAGCTCAATGCTAAACC 3΄ | AF380300 | 1482 | 494 | 55.4 |
| GPO3 | fw 5΄AAAGCCAGTGTCGCCG 3΄  rev 5΄TCAAGCAAGAAACTCAATTTTGATGCT 3΄ | KF032055 | 1539 | 513 | 57.1 |
| pSR7 |  | JQ388479 | 1488 | 496 | 56.3 |

* number of translated amino acids in the open reading frame

**Table S2: Molar absorption coefficients of products generated during oxidation.**

|  | **λ_max_ [nm]** | **ε_λmax_ [M^-1^ cm^-1^]** | **References** |
| --- | --- | --- | --- |
| **Monophenols** | | | |
| Tyramine | 480 | 3300 | 1 |
| (±)-Octopamine | 487 | 2140 | this work |
| Tyrosol | 399 | 1220 | this work |
| Phenol | 410 | 1623 | 1 |
| L-Tyrosine | 475 | 3600 | 1 |
| D-Tyrosine | 475 | 3600 | 1 |
| L-Tyrosine methyl ester | 476 | 7190 | this work |
| D-Tyrosine methyl ester | 476 | 7190 | this work |
| **Diphenols** | | | |
| Catechol | 410 | 1623 | 1 |
| 4-Methylcatechol | 400 | 1638 | 1 |
| Dopamine | 480 | 3300 | 1 |
| TBC | 400 | 1200 | 1 |
| Caffeic acid | 495 | 2062 | 1 |
| L-DOPA | 475 | 3600 | 1 |
| DOPAC | 453 | 1000 | this work |

**Table S3: Percentage of identity between produced isozymes and prototype sequences.**

| **Compared PPOs** | **Nucleotides %** | **Amino acids %** |
| --- | --- | --- |
| APO5 vs *Md*PPO1 | 99.93 | 100 |
| PPO2 vs *Md*PPO2 | 91.86 | 91.7 |
| GPO3 vs *Md*PPO3 | 97.92 | 97.9 |

**Table S4: Percentage amino acid sequence identity between each *Md*PPO1-3.**

| **PPO** | ***Md*PPO1** | ***Md*PPO2** | ***Md*PPO3** |
| --- | --- | --- | --- |
| *Md*PPO1 | x | 56.8 | 73.9 |
| *Md*PPO2 |  | x | 54.6 |
| *Md*PPO3 |  |  | x |

**Table S5: Characteristics of *Md*PPO1-3.**

| **PPO** | **Accession** | **Size**  **[bp]** | **Amino acids** | **MW^(a)^**  **[kDa]** | **MW_GST_^(b)^**  **[kDa]** | **MW_HRV3C_^(c)^**  **[kDa]** |
| --- | --- | --- | --- | --- | --- | --- |
| *Md*PPO1 | LT718522 | 1515 | 505 | 56.3 | 82.8 | 56.4 |
| *Md*PPO2 | LT718523 | 1485 | 495 | 55.3 | 83.7 | 57.3 |
| *Md*PPO3 | LT718524 | 1521 | 507 | 56.6 | 83.9 | 57.5 |
| ^(a)^MW: molecular weight of the native (latent) polyphenol oxidase (without signal peptide) | | | | | | |
| ^(b)^MW_GST_: molecular weight of the fusion protein | | | | | | |
| ^(c)^MW_HRV3C_: molecular weight of the expressed protein after removal of GST | | | | | | |

# Figures


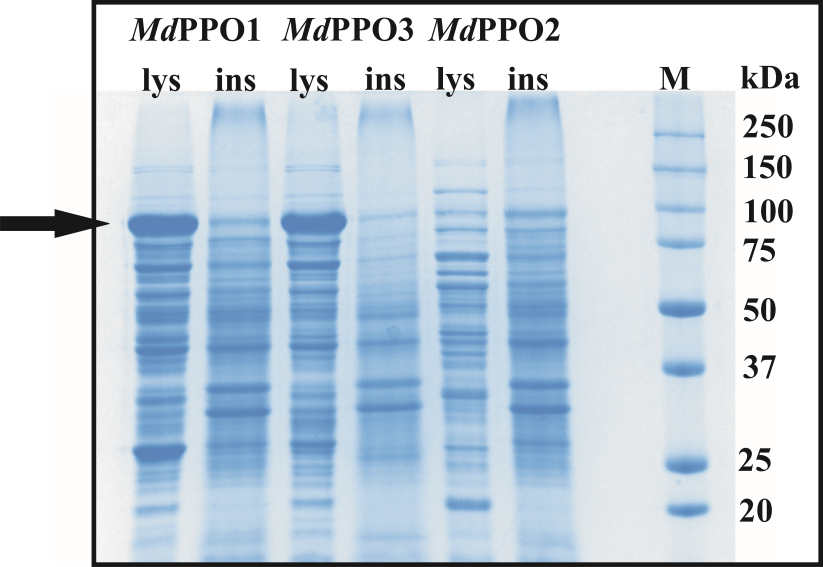


**Figure S1:** **Soluble (lys) and insoluble (ins) fractions from the heterologous expression of the *Md*PPO1-3**. The arrow indicates the position of the target protein.


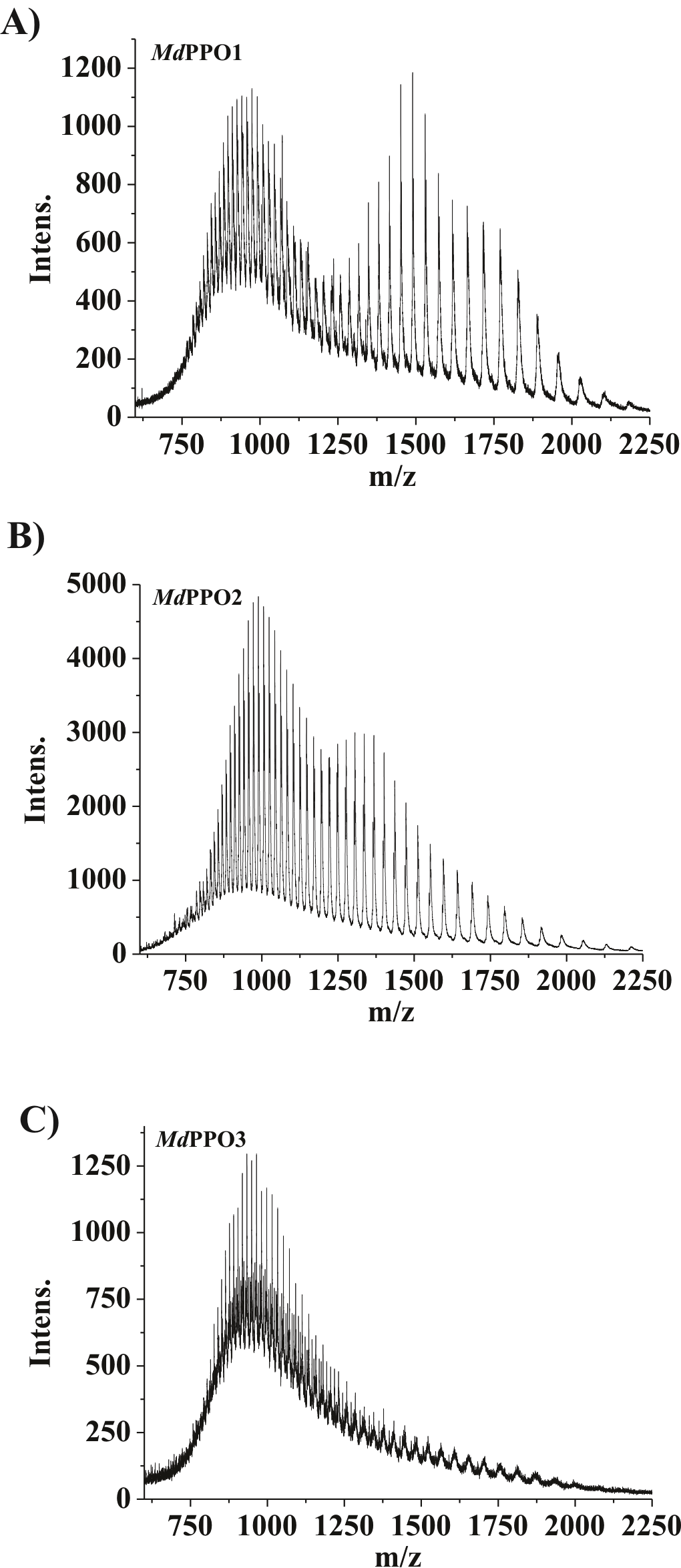


**Figure S2:** **Positive mode ESI-QTOF mass spectra.** Entire mass spectra of acidified samples of purified recombinant latent *Md*PPO1-3.

**
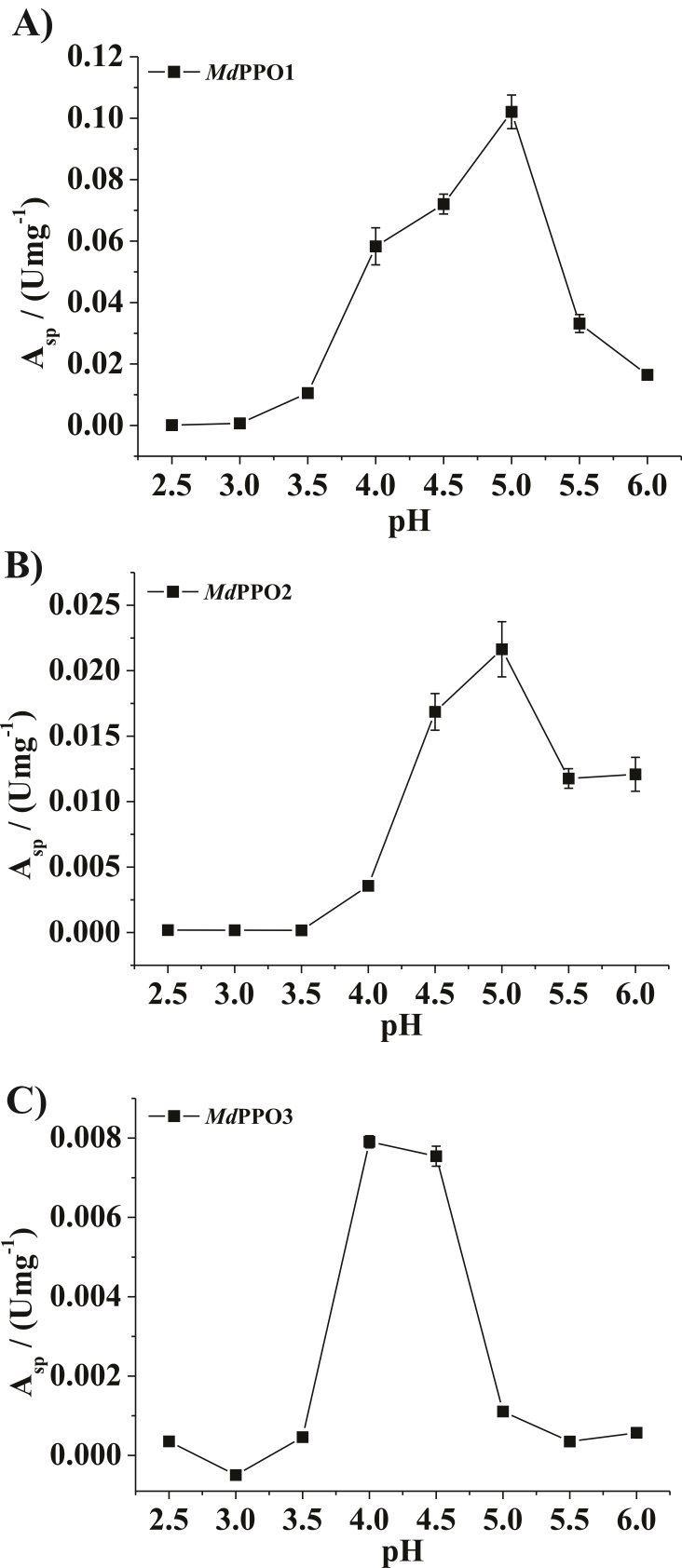
**

**Figure S3: Specific activity of the three *Md*PPO1-3 on 3 mM tyramine induced through acidic pH.** The error bars represent ± one standard deviation.

**
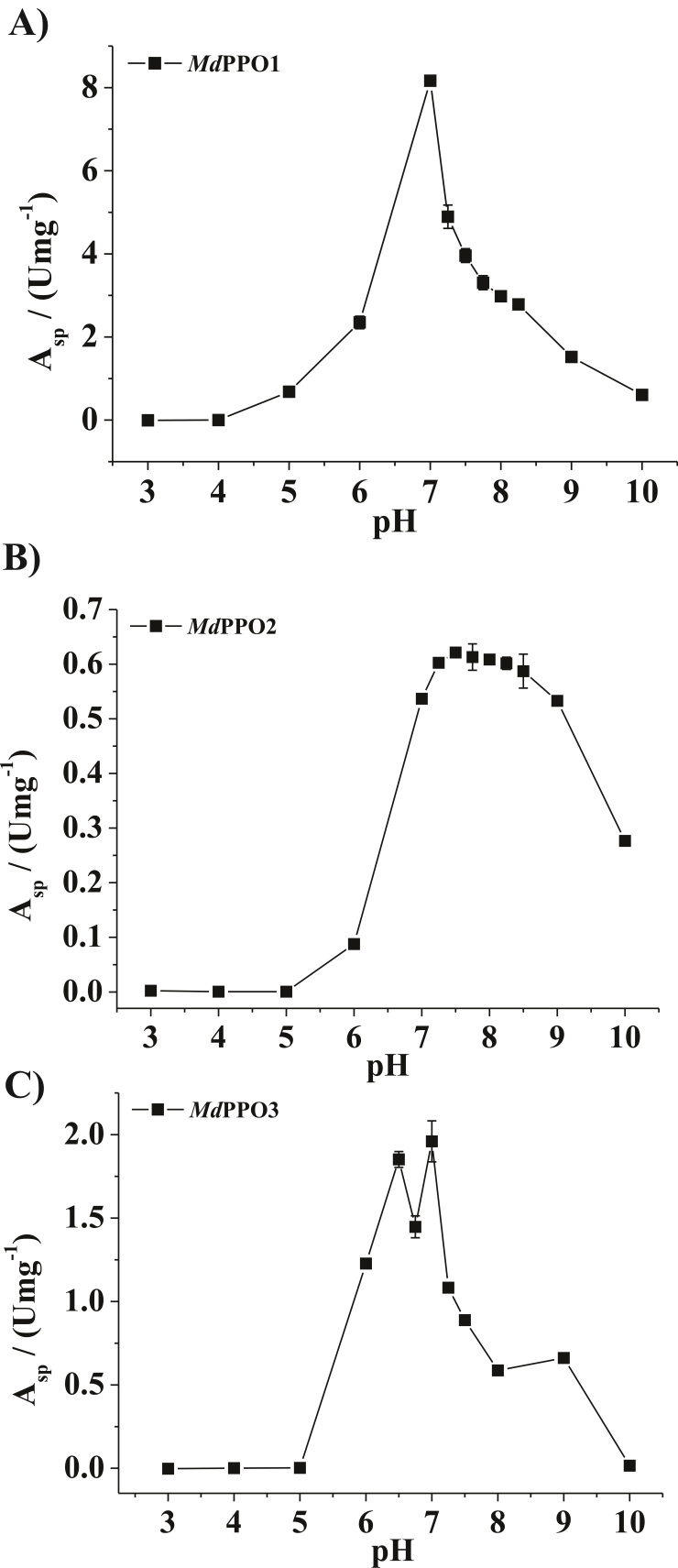
**

**Figure S4: pH optimum for activity of *Md*PPO1-3 with tyramine and SDS as the activating agent**. The activator SDS was used at the priority determined optimal concentration, which was 3 mM, 2 mM and 4 mM for *Md*PPO1, *Md*PPO2 and *Md*PPO3, respectively. The error bars show ± one standard deviation.

**
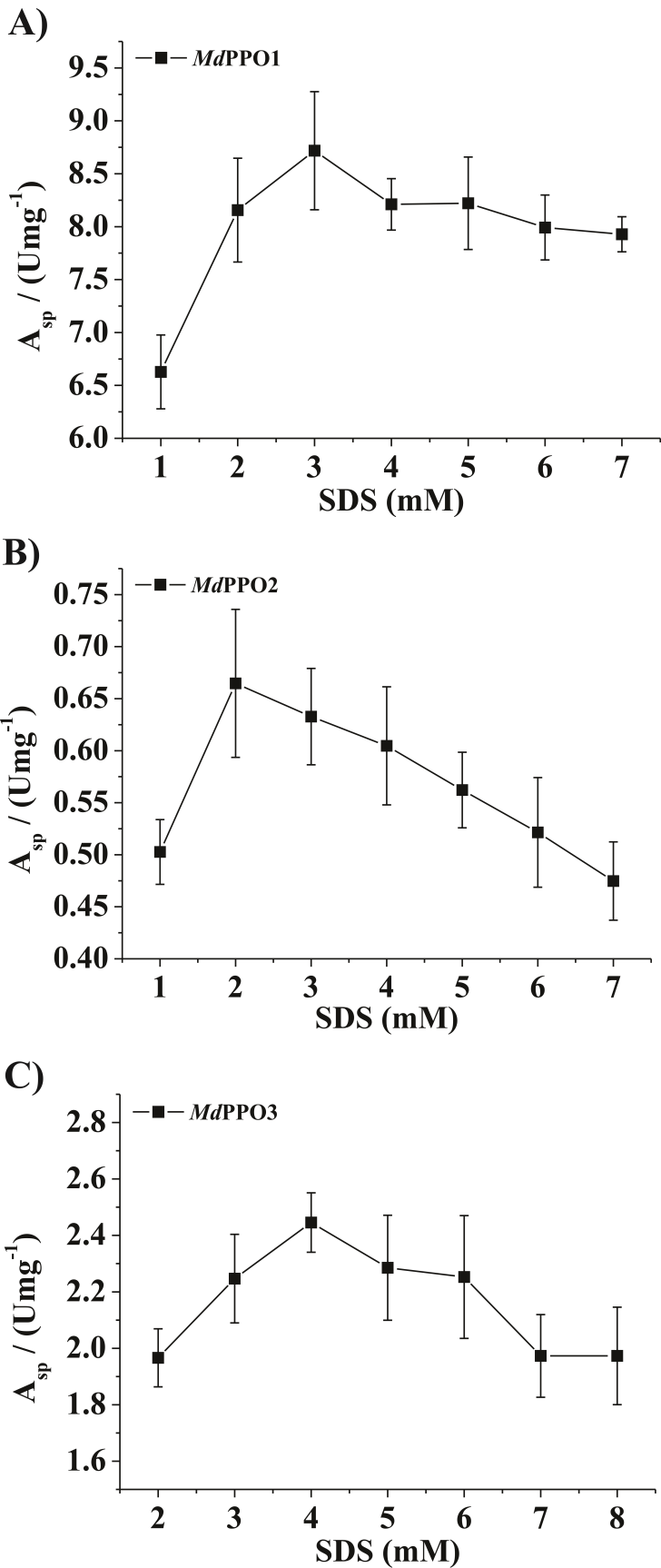
**

**Figure S5. SDS optimum for activity induction of *Md*PPO1-3 with 3 mM tyramine.** The error bars indicate ± one standard deviation.

| **Monophenols** |  | |
| --- | --- | --- |
|  |  |  |
|  |  |  |
|  |  |  |
| **Diphenols** |  | |
|  |  |  |
|  |  |  |
|  |  | |

**Figure S6:** **Structures of monophenolic and diphenolic substrates.**

**
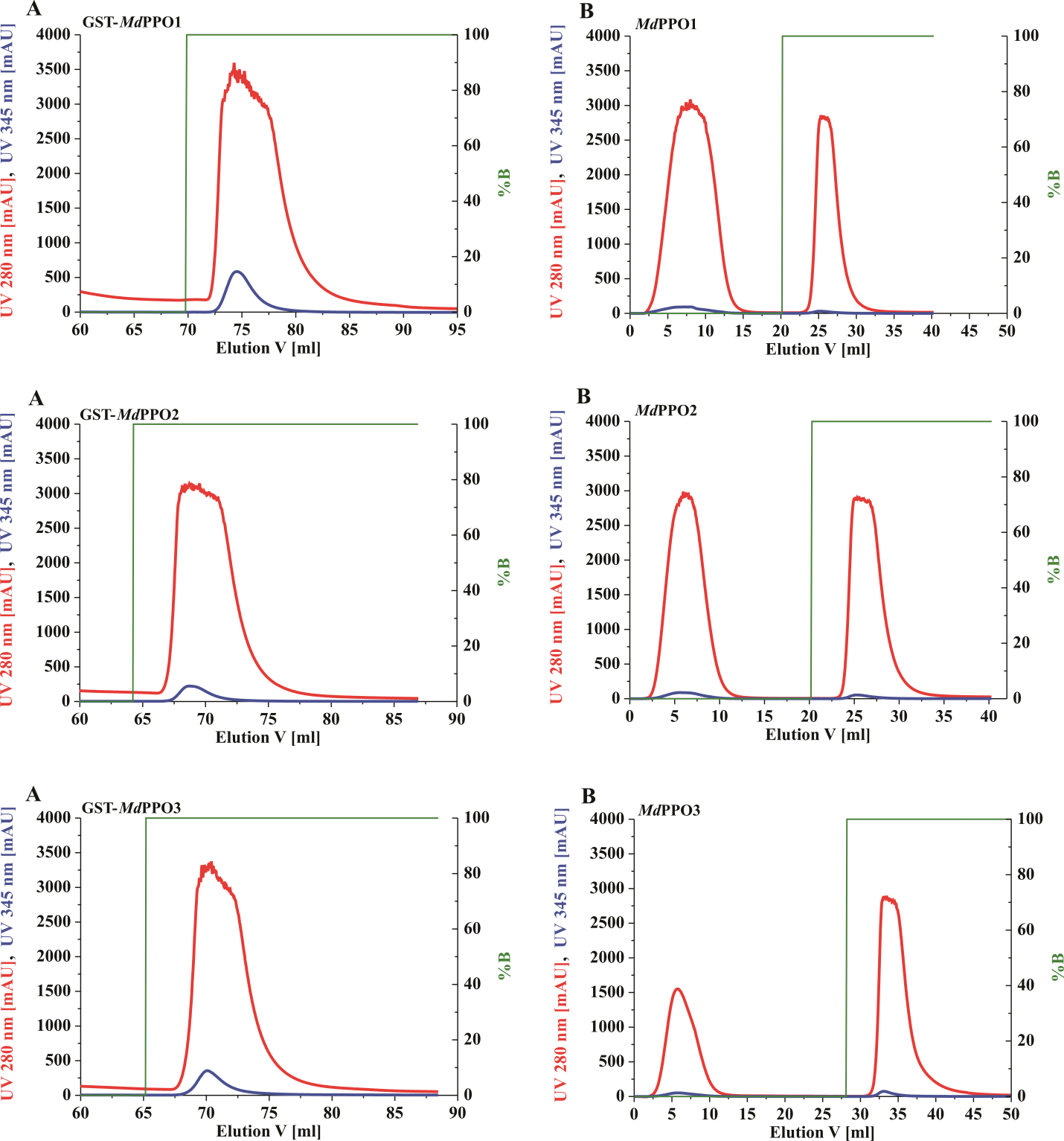
**

**Figure S7.** **Typical chromatograms of GST-affinity Purifications of *Md*PPO1-3** A) 1^st^ purification step of GST-*Md*PPO1-3: fusion protein elution, B) 2^nd^ purification step: separation of latent *Md*PPO1-3 (first peak) from GST and HRV3C (second peak).

**
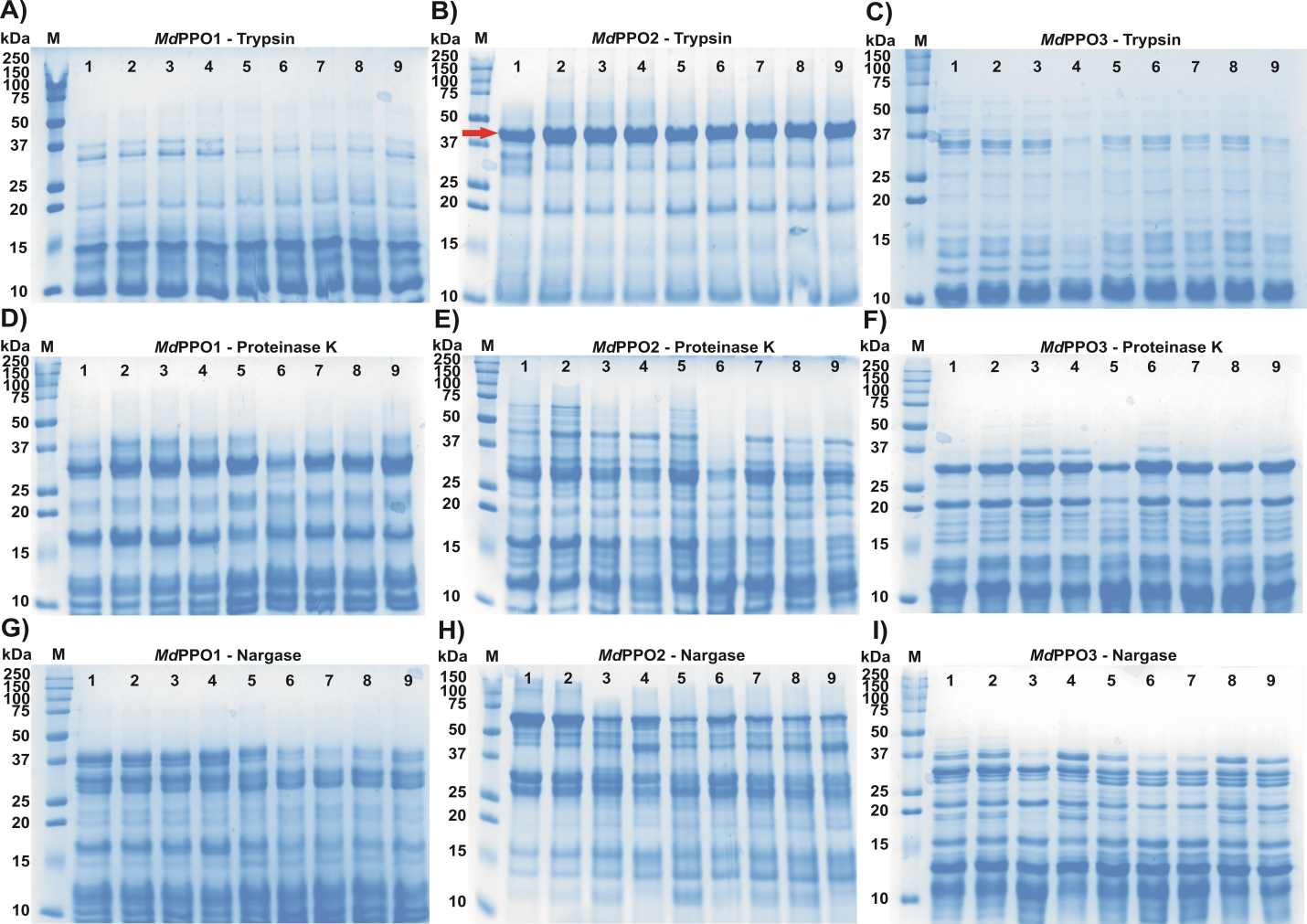
**

**Figure S8. Proteolytic activation of the latent *Md*PPO1-3: SDS – PAGE** The gels present the proteolytic activation of the latent *Md*PPO1-3 with three different proteases (A - C) trypsin, (D – F) proteinase, (K and G – I) Nagarse at different molar ratios of protein - protease and different reaction times. 1) 1:240 for 1 min, 2) 1:240 for 2 min, 3) 1:240 for 4 min, 4) 1:240 for 10min, 5) 1:120 for 1 min, 6) 1:120 for 2 min, 7) 1:120 for 3 min, 8) 1:120 for 4 min and 9) 1: 120 for 7 min. Arrow indicates the position of the active enzyme in the case of *Md*PPO2 with trypsin.

**
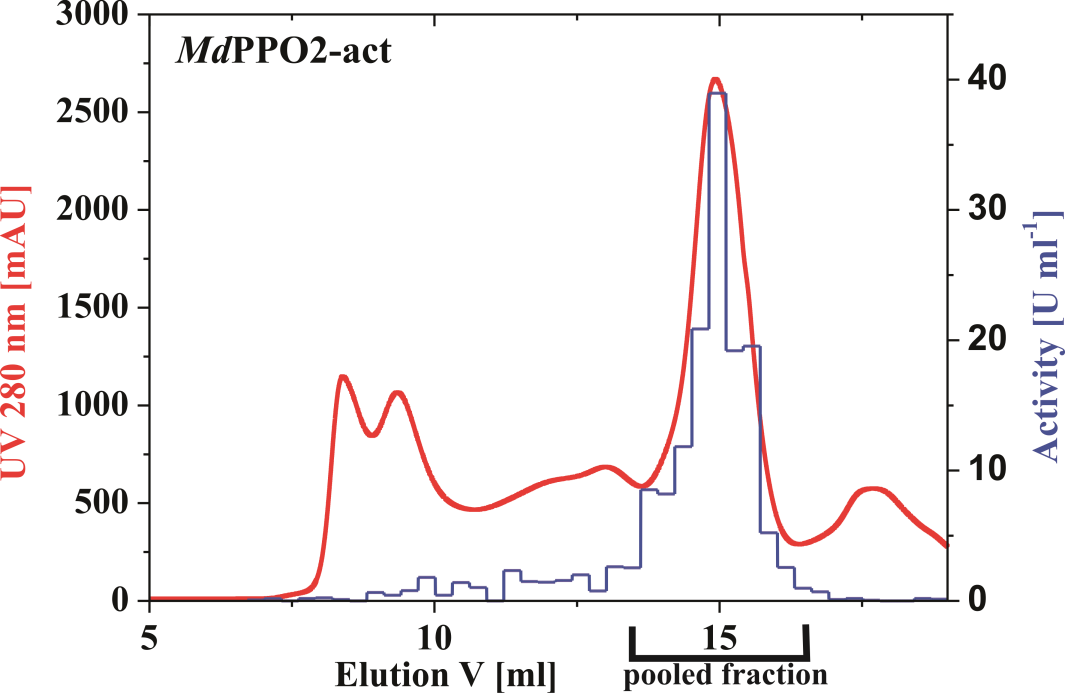
**

**Figure S9. Chromatogram of the active *Md*PPO2-act in Superdex 200 Increase.** Enzymatic assays for the active *Md*PPO2 have been performed with 1 mM dopamine in 50 mM Tris pH 7.5 at 480 nm.

**
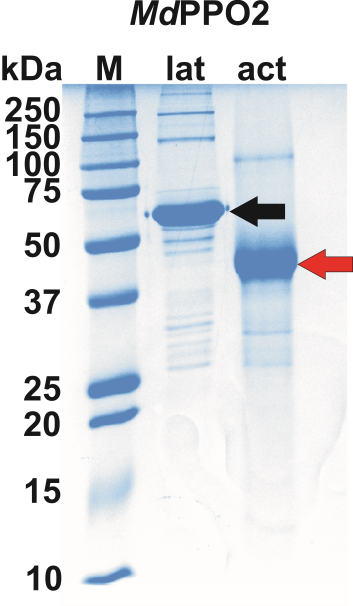
**

**Figure S10. Latent *Md*PPO2 and active *Md*PPO2-act SDS – PAGE.** The gel shows the latent *Md*PPO2(lat) black arrow and the active *Md*PPO2-act red arrow after the proteolytic activation with trypsin and the purification with the size exclusion column (Superdex 200 increase from GE Healthcare).


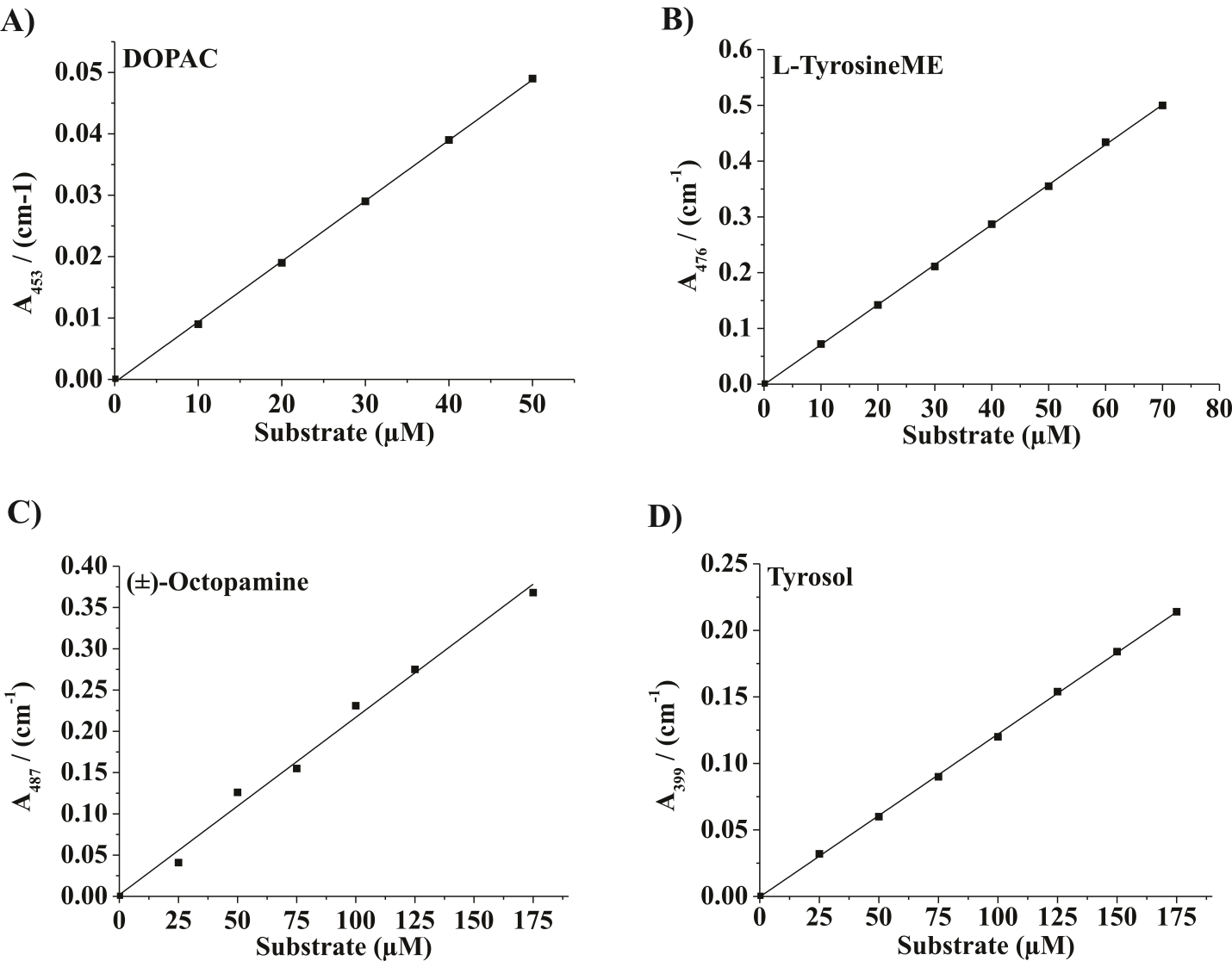


**Figure S11. Absorbances obtained by (hydroxylation and) oxidation of phenolic substrates.** The diphenols A) DOPAC (453 nm) and the monophenols B) L-Tyrosine methyl ester (476 nm), C) (±)-Octopamine (487 nm) and D) Tyrosol (399 nm) were measured.


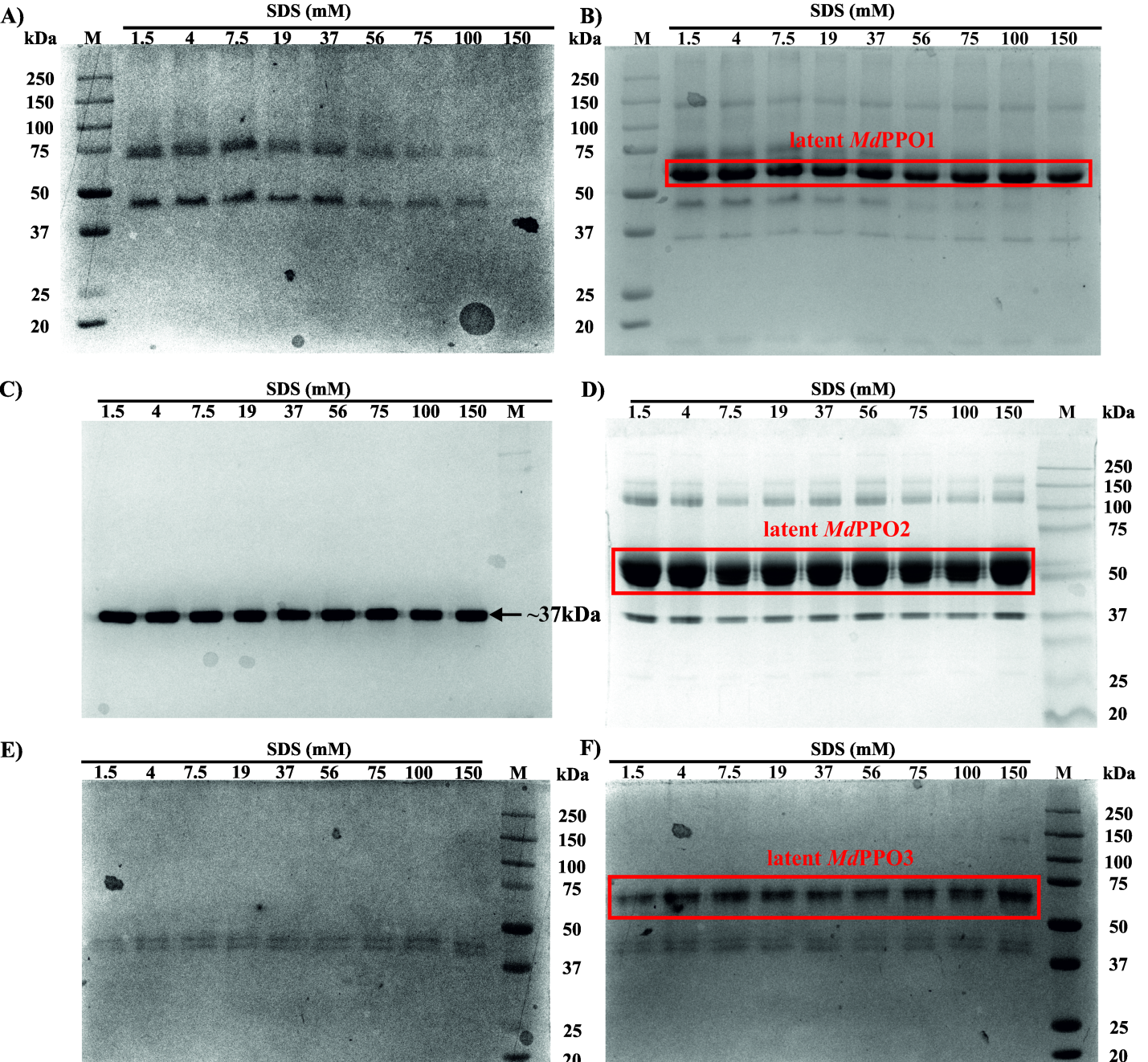


**Figure S12: Partially denaturating SDS-PAGE, activity staining for *Md*PPO1-3** A) *Md*PPO1 stained with 20 mM dopamine B) Duplicate of the respective gel *Md*PPO1 stained with Coomassie brilliant blue G-250. C) *Md*PPO2 stained with 20 mM dopamine D) Duplicate of the respective gel *Md*PPO2 stained with Coomassie brilliant blue G-250. E) *Md*PPO3 stained with 20 mM dopamine F) Duplicate of the respective gel *Md*PPO3 stained with Coomassie brilliant blue G-250.


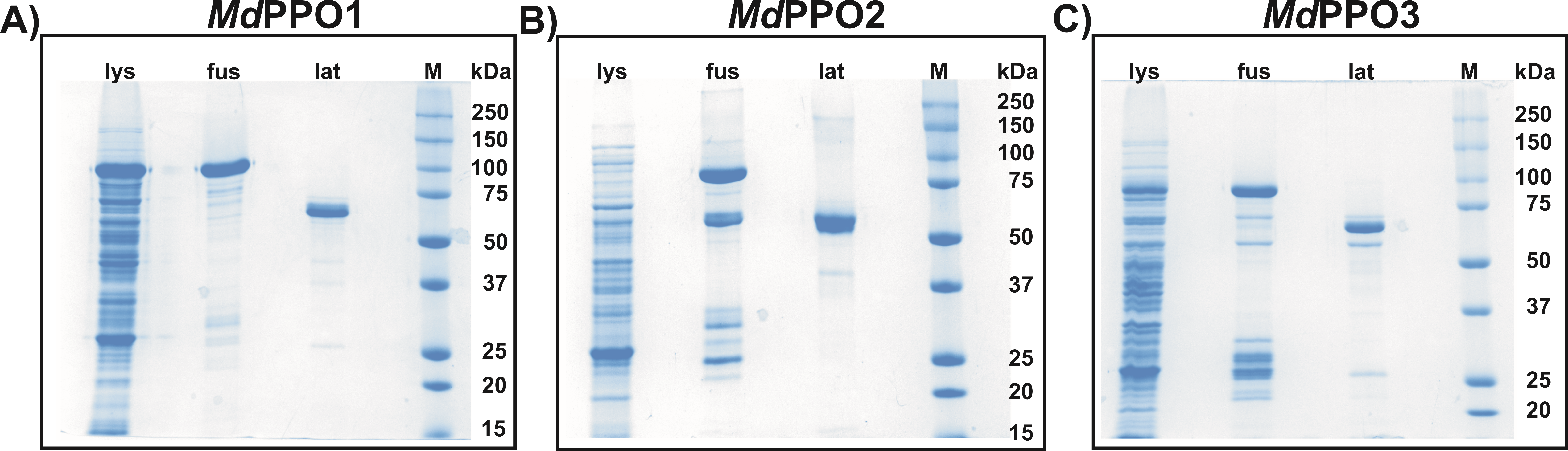


**Figure S13: Full-length gel of Figure 2**.

**Figure S14: Full-length gel of Figure 3**.

**References**

1. Muñoz, J. L*. et a*l. Calculating molar absorptivities for quinones: Application to the measurement of tyrosinase activity*. Anal. Bioche*m**. 35**1, 128–138 (2006).
